# Modulation of Epigenetic Targets for Anticancer Therapy: Clinicopathological Relevance, Structural Data and Drug Discovery Perspectives

**DOI:** 10.2174/138161213804581918

**Published:** 2013-02

**Authors:** Federico Andreol, Arménio Jorge Moura Barbosa, Marco Daniele Parenti, Alberto Del Rio

**Affiliations:** Department of Experimental Pathology, Alma Mater Studiorum - University of Bologna, Via S.Giacomo 14, 40126 Bologna, Italy

**Keywords:** Epigenetics, anticancer therapy, DNA methyltransferases, protein methyltransferases, demethylases, deacetylases, acetyltransferases, histone post-translational modifications, drug design, crystallography, small-molecule inhibitors.

## Abstract

Research on cancer epigenetics has flourished in the last decade. Nevertheless growing evidence point on the importance to understand the mechanisms by which epigenetic changes regulate the genesis and progression of cancer growth. Several epigenetic targets have been discovered and are currently under validation for new anticancer therapies. Drug discovery approaches aiming to target these epigenetic enzymes with small-molecules inhibitors have produced the first pre-clinical and clinical outcomes and many other compounds are now entering the pipeline as new candidate *epidrugs*. The most studied targets can be ascribed to histone deacetylases and DNA methyltransferases, although several other classes of enzymes are able to operate post-translational modifications to histone tails are also likely to represent new frontiers for therapeutic interventions. By acknowledging that the field of cancer epigenetics is evolving with an impressive rate of new findings, with this review we aim to provide a current overview of pre-clinical applications of small-molecules for cancer pathologies, combining them with the current knowledge of epigenetic targets in terms of available structural data and drug design perspectives.

## 1 INTRODUCTION 

The term epigenetics currently refers to the mechanisms of temporal and spatial control of gene activity that do not depend on the DNA sequence, influencing the physiological and pathological development of an organism. The molecular mechanisms by which epigenetic changes occur are complex and cover a wide range of processes including paramutation, bookmarking, imprinting, gene silencing, carcinogenesis progression, and, most importantly, regulation of heterochromatin and histone modifications [1]. At a biochemical level, epigenetic alterations in chromatin involve methylation of DNA patterns, several forms of histone modifications and microRNA (miRNA) expression. All these processes modulate the structure of chromatin leading to the activation or silencing of gene expression [2-6]. More specifically, the chromatin remodeling is accomplished by two main mechanisms that concern the methylation of cytosine residues in DNA and a variety of post-translational modifications (PTMs) occurring at the N-terminal tails of histone proteins. These PTMs include acetylation, methylation, phosphorylation, ubiquitylation, sumoylation, glycosylation, ADP-ribosylation, carbonylation, citrullination and biotinylation [7,8]. Among all PTMs for example, histone tails can have its lysine residues acetylated, methylated or ubiquitilated; arginine can be methylated; serine and threonine residues can be phosphorylated [9-17]. These covalent modifications are able to cause other PTMs and the ensemble of this cross-talk is known as the *histone code,* which can be positively or negatively correlated with specific transcriptional states or organization of chromatin [11,18-20]. The fine regulation of histone PTMs and DNA methylation is controlled and catalyzed by many different classes of enzymes whose existence and functions have been elucidated with an extraordinary progression in the last decade [12,20-25]. Epigenetic modifications are reversible nuclear chemical reactions that are due to enzymes able to exercise opposing catalytic effects [5,20]. Along with metabolism [26-28] and regulation of the immune system [29,30], epigenetic changes are at the limelight of cancer research. Many studies have found that alterations in the epigenetic code may contribute to the onset of growth and progression of a variety of cancers [20,21,23,31-41]. For this reason, these enzymes are attractive therapeutic targets for the development of new cancer therapies [3,42-45]. 

In this review we aim to present and discuss the relationship of the available information on epigenetic targets related to cancer pathologies and their structural data describing also the perspective for considering these enzymes as new targets for anticancer drug discovery initiatives. 

## 2 EPIGENETIC IN CANCER DISEASES 

**Fig. (1):**
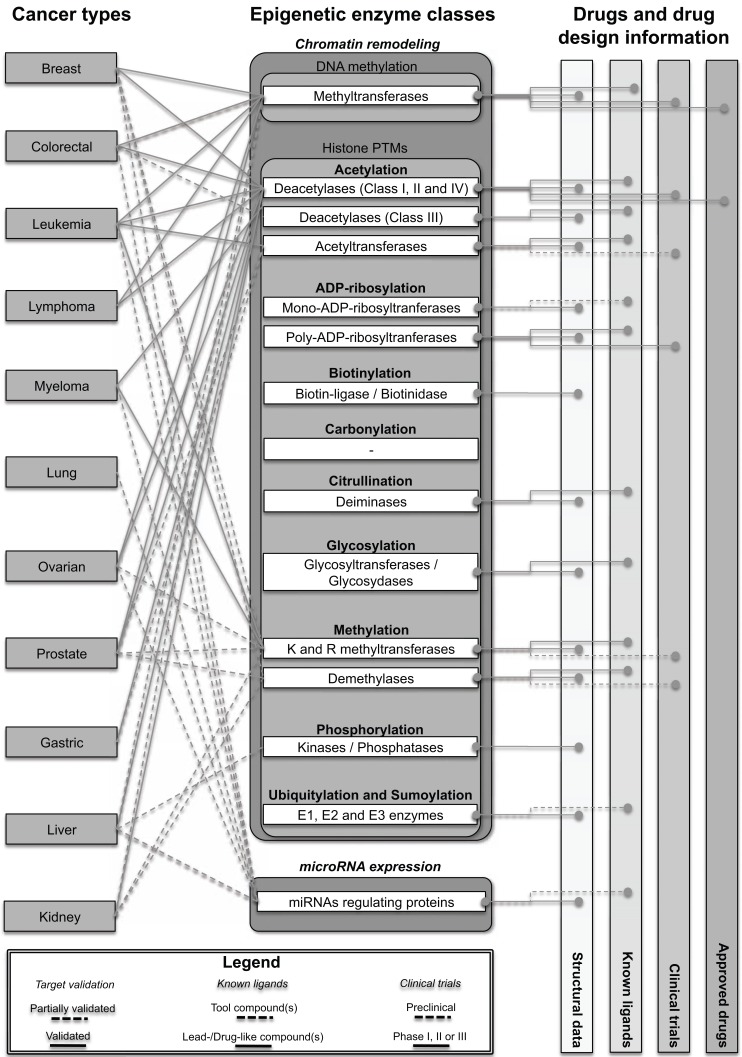
Connections between classes of epigenetic targets with cancer diseases and drug discovery information. Known ligands, clinical trials and approved drugs refer to cancer therapies with mechanisms of action directly related to epigenetic targets. Clinical trials and approved drugs have been recently reviewed in [46].

Although in the last decade several cancer pathologies have been associated to specific epigenetic changes, the way in which epigenetic modifications are regulated is still largely unknown. In this section we describe the current knowledge linking various cancer types with epigenetic targets, considering that demonstrated cause-consequence might not necessarily indicate that these targets are validated for anticancer drug design purposes. In (Fig. **1**) we summarized the connections between the most important cancer diseases and the various classes of epigenetic targets, associating them to relevant drug discovery information. 

### Breast Cancer 

Epigenetic alterations such as DNA methylation and chromatin remodeling play a significant role in breast cancer development and, although extensive research has been done, the causes, mechanisms and therapies of breast cancer are still to be fully elucidated [47-50]. Epigenetic changes in different classes of this type of cancer have been studied, including: estrogen receptor positive (ER+), that are estrogen-level dependent; estrogen receptor negative (ER-), whose tumor cells are not responsive to estrogen thus resistant to antiestrogenic drugs such as tamoxifen and aromatase inhibitors; progesterone receptor (PR); and human epidermal growth factor 2 (HER2)-related cancers [49,51-58]. A number of genes has been identified to be aberrantly methylated in breast cancer and their number is rapidly growing [48,56,59]. Likewise, altered expression of micro RNAs has been found to regulate key genes in the development of breast cancer [59-62]. Biological rationales for breast cancer therapies have been deeply studied by inhibiting DNA methyltransferases (DNMT) and histone deacetylases (HDAC) proteins. Furthermore, several epigenetic-based synthetic drugs, which can reduce DNA hypermethylation and histone deacetylation, are undergoing preclinical and clinical trials [49,57,63-65]. These *epidrugs *[55,66] are a promising strategy for breast cancer therapies as they could restore the estrogen receptor α (ERα) activity in ER- cancer patients, reactivating cancer cell growth in an estrogen-dependent manner resulting sensible to antiestrogenic drugs [51,52,55,58,67-69]. Additional studies include epigenetic targets such as methyltransferases [70,71] which are currently in the spotlight of drug discovery programs, not only for breast cancer but also for a number of other conditions [24,41,72-74]. Besides, dietary components like complementary and/or alternative medicines from green tea, genistein from soybean, isothiocyanates from plant foods, curcumin from turmeric, resveratrol from grapes, and sulforaphane from cruciferous vegetables, have been studied for their ability to target the epigenome in relation to breast cancer; nevertheless their mechanisms of action are still poorly characterized [49,66,75-77]. 

### Colorectal Cancer 

Extensive loss of DNA methylation has been observed in colon cancer cells almost 30 years ago [78]. Epigenetic abnormalities associated with colorectal cancer (CRC) have been, since then, intensively studied to identify the methylation patterns appearing at the various stages of colorectal cancer progression [79-83]. Frequent targets of aberrant methylation processes and CRC markers have been recently reviewed [84-86]. Epigenetic changes in colorectal cancer have been studied in relation to chromosomal instability [81-83,87], inflammation and microenvironmental role of gut microbiota [88], genetic polymorphism [89] and nutraceuticals [90-92]. In addition, epigenetically modified miRNAs have also been found to play a role in CRC [93,94]. The silencing of some miRNAs is associated with CpG island hypermethylation. The aberrant hypermethylation of two miRNAs (miR-34b/c and miR-148a) has been reported as a possible early screening and disease progression markers.[95] Further investigations identified 35 miRNAs related to colon cancer that were epigenetically silenced and revealed 162 molecular pathways potentially altered by eight methylated/downregulated miRNAs in CRC [61,96]. As major pathways of colorectal carcinogenesis are tightly connected to epigenetic changes, growing evidence shows that the risk of CRC can be influenced by lifestyle and environmental factors [91]. For instance, flavonoids and folates in a human diet have been shown to alter DNA methylation and modify the risk of human colon cancer and cardiovascular diseases, even though these mechanisms are yet to be ascertained.[90,97] Additional researches on the effects of nutraceuticals on epigenetic changes in the intestinal mucosa promise to be relevant for preventive and therapeutic interventions [91]. Pharmacological inhibition of Class I and Class II HDACs and the emerging role of Class III (in particular Sirt1) have been studied for their capacity to induce growth arrest, differentiation and apoptosis of colon cancer cells *in vitro* and *in vivo* [98-101]. Consequently, several clinical trials were initiated to repurpose compounds for CRC that were already approved or were in late-stage trials for the treatment of hematopoietic and solid tumors. 

### Hematological Malignancies 

DNA and histone post-translational modifications have been demonstrated to be associated with several mutations in epigenetic targets for different hematologic malignancies [102]. 

In leukemias the role of different epigenetic enzymes has been investigated mainly for acute promyelocytic leukemia (APL) [103,104] and acute myeloid leukemia (AML) [103]. Biological players that have been studied for clinical applications include deacetylases [32,105-108], DNA and histone methyltransferases [32,35,103,104,109-125] and miRNA [104,119,126,127]. Besides APL and AML, further data have been collected for leukaemogenesis, including transforming factors and epigenetic alterations [106,111,128-131]. Several small organic molecules have been proposed for clinical use in different leukemia pathologies. Histone deacetylase inhibitors (HDACi) such as Panobinostat (LBH589), Belinostat (PXD-101), 4SC-202 and AR-42 are currently in clinical trials for the treatment of chronic myeloid leukaemia (CML), AML and chronic lymphocytic leukemia (CLL) [35,104-107,132]. A considerable interest in using HDACis is the study of combined regimens with other agents that can enhance cancer cell lethality. Among those agents there are cyclin-dependent kinase and tyrosine kinase inhibitors as well as Hsp90 and proteasome inhibitors [133,134]. Histone methyltransferases have also been the object of drug design approaches for leukemias. For instance, disruptor of telomeric silencing 1-like (DOT1L) has been discussed as a potential target of for the mixed-lineage leukemia (MLL) fusions. The potent SAM-competitive DOT1L inhibitor EPZ004777 was reported together with clinical implications for the personalized treatment of such an aggressive form of leukemia [109,110,113]. In addition, the structure of the newly developed inhibitor GSK2816126 targeting EZH2 for the treatment of AML was unveiled at the 2012 American Association for Cancer Research (AACR) annual meeting [135]. This compound was found to abrogate histone overmethylation, and the treatment of cell cultures and laboratory animals with this compound demonstrated a reduced proliferation of tumor cells. 

The interest in modulating epigenetic enzymes is also rising in the treatment of lymphomas and myelomas, particularly as combination therapies. For instance, HDACi and DNMT inhibitors have been tested for the treatment of aggressive non-Hodgkin’s Lymphomas (NHLs) [136-145]. 

Overall, pre-clinical and clinical studies in hematological malignancies are presently focused on histone deacetylases and DNA methyltransferases, but growing evidence points to the development of therapies that are directed to other classes of epigenetic enzymes, especially histone methyltransferases [146]. 

### Lung 

Epigenetic changes in lung cancers contribute to cell transformation by modulating chromatin structure and specific expression of genes; these include DNA methylation patterns, covalent modifications of histone and chromatin by epigenetic enzymes, and micro-RNA. All these changes are involved in the silencing of tumor suppressor genes and enhance the expression of oncogenes [147-152]. Genome-wide technologies and bioinformatics studies demonstrated that global alterations of histone patterns are linked to DNA methylation and are causal in lung cancer [153,154]. These techniques were also used for the prediction of specific miRNAs targeting the epidermal growth factor receptor (EGFR) in lung cancer [155]. Many genes were found to be silenced by methylation promoters in lung cancers in response to radiation stimuli [156]. DNA methylation patterns may also predict early recurrence of stage I non-small-cell lung carcinoma (NSCLC) [149]. 

As lung cancer is the major cause of cancer death worldwide and the five-year survival is extremely poor, the need of more effective therapeutic agents is of utmost importance [154]. In particular, NSCLCs are relatively insensitive to chemotherapy when compared to small cell carcinomas, so efforts are now directed to the study of the epigenetic changes occurring in these type of cancers and in pulmonary hypertension [154,157]. Restoration of the expression of epigenetically silenced genes with new targeted approaches and combined therapy with azacitidine and entinostat, as well as DNMTi and HDACi, were investigated in phase I/II trials for the treatment of NSCLCs [158-160]. 

### Ovarian 

Ovarian cancer is the most lethal gynecologic cancer. In advanced ovarian and endometrial carcinomas, current therapies that are initially responsive, evolve to a fully drug-resistant phenotype [161,162]. Among the factors that contribute negatively to the progression and therapeutic resistance against ovarian and endometrial cancer, there are several genetic mutations and epigenetic anomalies which are frequent in both malignancies [163-168]. Epigenetic changes include aberrant DNA methylation, atypical histone modifications and unregulated expression of distinct microRNAs, resulting in altered gene- expression patterns favoring cell survival [162, 169,170]. 

As for other cancer diseases, the therapeutic intervention aimed at reversing oncogenic chromatin aberrations have been primarily studied with DNMTs and HDACs inhibitors [162,168,171,172]. In addition, epigenetic phenomena, in which post-transcriptional gene regulation by small non-coding microRNAs is relevant, have also been investigated. Targeting of specific miRNAs has been performed using antagomir oligonucleotides for both mechanistic studies and investigation of possible *in vivo* therapeutic applications [170]. 

### Prostate 

Prostate cancer is one of the most commonly diagnosed cancers in men. A rapid increase of the incidence for this cancer is expected as the male population over the age of fifty is growing worldwide. In this cancer, epigenetic alterations appear earlier and more frequently than genetic mutations. Multiple genes silenced by epigenetic alterations have been identified [173]. Several reviews describing epigenetic changes in prostate cancer have been published recently [173-179]. Anti-cancer drug research has been stimulated by the fact that, for patients who are not cured by local treatment and have metastasis, neither androgen ablation nor chemotherapy can abrogate progression. For this reason, finding pharmacological strategies aimed to control prostate cancer initiation and disease progression is still a medical challenge. Several studies connecting prostate cancer and epigenetics include insights into: hypermethylation and hypomethylation patterns [180-184], involvement of histone modifiers such as HDACs, histone acetyltransferases (HATs), protein lysine methyltransferase (PKMTs) [185-187], multicomponent epigenetic regulatory complexes [188-191], new molecular biomarkers and therapeutic implications [192,193] and prevention with dietary components [183,194,195]. Preclinical evidence involving the epigenome as a key mediator in prostate carcinogenesis has entailed initial clinical trials with *epidrugs* such as HDACs inhibitors [174]. It is expected that future drugs could become useful for new combination regimens aimed at treating prostate cancer. 

### Gastric 

Gastrointestinal (GI) carcinogenesis causes some of the most common types of tumors worldwide, including esophagus, stomach, bowel, and anus. Even thought it has been recognized that the major reason for GI carcinogenesis resides in at least one genetic mutation that either activates an oncogene or inhibits the function of a tumor suppressor gene, recent data indicate that epigenetic abnormalities are critical in regulating benign tumorigenesis and eventual malignant transformation in gastorointestinal (GI) carcinogenesis [196-202]. In particular, aberrant histone acetylation regulated by HATs and HDACs have been linked to gastric cancer [196]. Epigenetic alterations have also been identified in presence of Epstein-Barr virus [203-205], while *Helicobacter pylori*, which constitutes a main cause of gastric cancer, was shown to reduce HDACs activity. These data suggest that pharmacological actions of HDACi in GI might be detrimental or beneficial depending on the clinicopathological context [206,207]. Despite the fact that various links between GI cancer and HATs and HDACs have been identified, comparing to other cancers, fewer progresses have been reported to treat GI carcinogenesis with *epidrugs*. A Phase I study has combined Vorinostat with radiotherapy in GI carcinoma [208]. This, as well as other studies, created foundations for additional initiatives to improve the therapeutic potential of HDACi and other epigenetic enzymes for GI tumors [196]. 

### Liver 

Hepatocellular carcinoma (HCC) originates from hepatocytes and is the most common liver cancer. Cancer rates and etiology of HCC vary considerably by age, gender, ethnic origin, lifestyle (in particular alcohol abuse [209]) and environmental pollution [210]. Other factors include the infection by hepatitis B and C virus (HBV and HCV) [211,212], exposure to aflatoxins, hypertension and diabetes [210,213]. Both genetic and epigenetic factors form the molecular basis of HCC. Epigenetic alterations may predispose to genetic changes and, *vice versa*, genetic changes may also initiate aberrant epigenetic modifications [210,213-216]. DNA methylation and various histone modifications, as well as RNA interference, have been reported as epigenetic events contributing to HCC development [210,215,217]. It should be remarked that the use of epigenetic biomarkers for detecting hepatocellular carcinoma has expanded the potential for non-invasive screening of high-risk populations [218]. However, the road to develop small-molecule compounds targeting epigenetic enzymes for HCC cancer treatment is at its beginning. Presently only HDACis have been studied for the treatment of HCC [217,219-221]. 

### Kidney 

Kidney cancer accounts for 2% of all adult cancer malignancies and the majority of them (80-85%) are renal cell carcinomas (RCCs) originated from the renal parenchyma. While the direct causes of this type of cancer are still vaguely defined, smoking and chemical carcinogens (e.g. asbestos and organic solvents) have been related to renal tumorigenesis [222]. Furthermore pathologies like obesity, hypertension and the use of antihypertensive medications, have been reported as risk-factors for RCCs [222,223]. Stepwise accumulation of DNA methylation has been observed by comparing normal renal tissues, renal tumor tissues and non-tumor renal tissues of patients with renal tumors [222]. These results highlighted that regional CpG patterns may participate in the early and precancerous stage of renal tumorigenesis. On the contrary, DNA hypomethylation does not seem to be a major event during renal carcinogenesis. DNA methylation alterations at a precancerous stage may further predispose renal tissue to epigenetic and genetic alterations, generating more malignant cancers and even determining the patient outcome [223]. At present there are few clinical trials of Phase I/II for testing inhibitors of HDACs (i.e. LBH-589 and Vorinostat) in advanced RCC [224,225]. 

## 3 STRUCTURAL DATA OF EPIGENETIC TARGETS 

The research aiming at developing new therapeutic anticancer strategies against epigenetic targets has flourished in the last years. Several review articles recently described rationales, targets, new drugs, approaches, novel compounds and methodologies [12,17,18, 20,22-25,35-41,65,226-229]. A large amount of these insightful articles have been dedicated to well established drug targets such as the histone deacetylases (HDACs) and DNA methyltransferases, and to the status of the development of small-molecule compounds [25,31,45,55,230,231]. However, despite the intensive research effort, molecular processes linking specific epigenetic targets to DNA-dependent biological functions, and their cause-consequence relationships, have been hard to elucidate. Beyond the fairly well characterized epigenetic processes of histone acetylation and methylation, many other PMTs require further biological elucidations, currently collected by many scientists conducting research on cancer epigenetics. In the next paragraphs, we describe classes and families of proteins that have been directly and/or indirectly associated to the modulation of the epigenetic code, taking into account their importance in cancer pathologies. We emphasize that structural information does not imply that these proteins can be considered validated drug targets for anticancer treatments, as other factors need to be considered. Figure **1** provides a graphical view of the information related to these targets. In the next sections we describe in tabular form the ensemble of structural data related to these targets, as suggested by the current state-of-the-art in the field. For additional information, the reader will be referred to other important reviews and articles in each relevant section. 

### Acetylation 

#### Class I, II and IV Deacetylases 

Histone Deacetylases (HDACs) contribute to the regulation of transcriptional activity by catalyzing the hydrolysis of acetyl-L-Lys side chains of histone and non-histone proteins in L-Lys and acetate. By restoring the positive charge of Lys residues, HDAC enzymes reverse the catalytic activity of histone acetyltransferases that will be described below. Deacetylation of histones alters the chromatin structure and represses transcription. Abnormal activity of these enzymes is implicated in several diseases, especially in cancer [20,23,31,98,107,136,159,232-238]. 

To date, 18 HDACs have been isolated in humans. They are organized into: class I (HDACs 1, 2, 3 and 8), class IIa (HDACs 4, 5, 7 and 9), class IIb (HDACs 9 and 10), class III (designated sirtuins SIRT1 to 7) and class IV (HDAC11). Class III enzymes are NAD^+^-dependent deacetylases that are catalytically distinct from other HDAC classes, thus they will be discussed in the next paragraph. X-ray crystal structures (Table **1**) are available for human HDACs (2, 3, 4, 6, 7, and 8) and for three HDAC-related deacetylases from bacteria, namely, histone deacetylase-like proteins (HDLP), histone deacetylase-like amidohydrolases (HDAH) and acetylpolyamine amidohydrolases (APAH). The first three-dimensional structure of an HDAC-related protein was the histone deacetylase-like protein (HDLP) from *Aquifex aeolicus* in complex with the inhibitors Tricostatin A and SAHA (Vorinostat) [239]. This data provided the structural basis for the catalytic mechanism and the inhibition of this family of enzymes, paving the way for the design of new bioactive molecules able to interfere with the deacetylation reaction. Several compounds targeting HDACs entered clinical trials in the last year and have been reviewed elsewhere [98,105,139,141,234,237,240-243]. These proteins belong to the open α/β folding class, with an eight-stranded parallel β-sheet sandwiched between α-helices. The active site consists of an extended and tight primarily hydrophobic tunnel with the catalytic machinery located at its end. During the deacetylation reaction the tunnel is occupied by methylene groups belonging to the substrate acetylated Lys, while the acetyl moiety binds a metal ion in the center of the active site. The deacetylase reaction requires a transition metal ion and, although the HDACs are typically considered Zn^2+^-containing enzymes, the metal ion in the active site, as demonstrated by the X-ray structure of HDAC8, can be substituted by Fe^2+^, Co^2+^ and Mn^2+^ [244]. This is consistent with the hypothesis that HDAC8 could function as a Fe^2+^-catalyzing enzyme *in vivo* (Table **1**) [245]. The overall fold of other recently crystallized HDACs is similar to the previously reported structures, even if several key features distinguish the various classes. A comprehensive review on these structural aspects has been published by Lombardi *et al* [246]. Besides the large number of non-mutated X-ray structures of the catalytic domain, often in complex with known inhibitors, three-dimensional structures of other HDAC domains have also been published. In particular, three structures of the zinc-binding domain of HDAC6 and two structures of the glutamine-rich domain of HDAC4, both responsible of protein-protein interactions and formation of large protein complexes, have been solved (Table **1**). In addition, the large number of complexes with point mutations in the catalytic domain, especially for HDAC8, HDAC4 and bacterial APAH, highlight the importance of some key residues in the binding of substrate and small-molecule inhibitors. 

#### Class III Deacetylases (Sirtuins) 

Sirtuins represent the class III family of histone deacetylases (HDACs). Structure and function of these proteins differ from other HDACs since sirtuins require NAD^+^ to catalyze the removal of an acetyl moiety from a Lys residue within specific protein targets, including histone tails. As seen in the previous section, this family of enzymes is largely conserved from bacteria to humans [264] and is involved in important physiological processes and disease conditions including longevity, metabolism and DNA regulation, cancer and inflammation [265,266]. In the last years, the three-dimensional structures of many sirtuin homologs have been solved by X-ray crystallography allowing a better understanding of the catalytic mechanism and specific structural features of this enzyme family (Table **2**). The first three-dimensional structure obtained was a Sir2 homolog from *A. Fulgidus* complexed with NAD^+^ [267]. This structure provided the first insights into the structural features and catalytic mechanism of sirtuins. Afterward, further details were provided about the active site characteristics, deacetylation reaction and inhibitors/substrate binding as a result of several X-ray structures complexed with different substrates. Examples include: p53 peptides or histone H3/H4 peptides, and different reaction intermediates, like 2-O-acetyl-ADP-ribose (see Table **2** for details). The inhibition mechanism of the endogenous regulator nicotinamide, a key step in the development of new sirtuins effectors, was also studied [268,269]. Although a large number of synthetic sirtuins inhibitors and activators are described in literature, only one co-crystal structure reports an inhibitor, Suramin, showing the structural basis for inhibitor binding and allowing the rational design of new and more potent compounds [270]. Several bacterial Sir2 structures and human Sirt2, Sirt3, Sirt5 and Sirt6 are available whereas no structures exist at present for Sirt1, Sirt4 and Sirt7 (Table **2**). All these PDB entries contain the catalytic domain, formed approximately by 270 residues, and variable N-terminal and C-terminal regions. The catalytic core of sirtuins is conserved among the various isoforms; it is formed by a large Rossman-fold domain, present in many NAD^+^-binding proteins, and a small zinc-binding domain. A number of flexible loops bring together the two domains to form a large groove that accommodates both cofactor and substrate. A review on sirtuins is also part of the current journal issue [271]. 

#### Acetyltransferases 

Histone acetyltransferases (HATs) utilize acetyl-CoA (AcCoA) as cofactor and catalyze the transfer of an acetyl group to the ε-amino group of Lys side chains of histone proteins to promote gene activation. Two major classes of HATs have been identified, Type-A and Type-B. Type A HATs can be classified into three families, based on sequence homology and conformational structure: GNAT, p300/CBP, and MYST [288]. These proteins are able to acetylate multiple sites within the histone tails, and also additional sites on the globular histone core. Type-B HATs are mostly cytoplasmic and acetylate newly synthesized histones, H3 and H4, at specific sites prior to their deposition into chromatin. Proteins in this class are highly conserved and share some sequence identity with HAT1 from yeast, the most studied member of this family [289]. Three-dimensional structures of different HATs reveal a structurally conserved catalytic core domain that mediates the binding of the cofactor AcCoA and non-conserved N-terminal and C-terminal domains specific for each protein to mediate histone binding [290]. HAT proteins are often associated with other subunits in large multiprotein complexes playing important roles in modulating enzyme recruitment, specificity and activity. The combination of these subunits contributes to the unique features of each HAT complex. For example, some subunits have domains such as bromodomains, chromodomains, Tudor domains and PHD fingers that cooperate to the enrollment of HAT complexes to the appropriate location in the genome by means of modified histone tail recognition [291]. To date, several three-dimensional structures obtained both from X-ray crystallography or NMR are available for human and bacterial HATs; among these, only few structures report the full-length protein, while others describe only specific domains and their interactions with other subunits and/or substrate (see Table **3**). 

A growing interest on novel drug design initiatives is currently focused on the primary readers of the histone code, the histone binding domains (HBDs). Notable HBDs are the Bromodomain (BD) proteins, which are structurally small and evolutionary conserved modules that bind acetyl-Lys and are part of larger BCPs (bromodomain containing proteins) [344]. These modules are frequently found in HATs as well as members of the histone methyl-transferase (HMT) family and ATP-dependent remodeling enzymes [19,345]. At least 56 BDs are encoded in the human genome and translated in 42 different known proteins whose structures, for half of them, have been determined by X-ray crystallography [344,346]. The research focused on inhibition of BDs has been stimulated by the discovery of two potent compounds (I-BET762 and JQ1) with *in vivo* efficacy in murine models of NUT (nuclear protein in testis) midline carcinoma, as well as AML and severe immune inflammation [347-350]. Other recent works show the application of fragment-based drug discovery techniques for the identification of new BD inhibitors [316,347,348,351,352] in addition to evidence that the pharmacological inhibition of BET (bromodomains and extra terminal domain) family proteins leads to rapid and potent abrogation of *MYC* gene transcription [353]. 

### Methylation 

#### Histone Methyltranferases 

Protein methyltransferases (PMTs) are a group of histone-modifying enzymes belonging to the large number of coded PTMs [73,354,355]. Currently two different classes of PMTs are recognized: protein lysine methyltransferases (PKMT) and protein arginine methyltransferases (PRMT), which are encoded in 51 and 45 genes [356], respectively. PMTs emerged recently as new important targets for cancer therapy since they were found to be overexpressed or repressed in several types of cancer [73]. PKMTs can mono-, di- or tri-methylate target Lys residues, whereas PRMTs are able to mono- or di-methylate the histone Arg residues [41,72,73, 191,355,357-363]. PKMTs share a conserved active site in the so-called SET (Su(Var)3-9, Enhancer of zeste, Trithirax) domain. The only known exception is the DOT1L, which has PKMT activity without having the SET domain in its structure. DOT1L also shares a higher homology towards PRMTs and is often reported in the PRMTs family tree diagrams as having a PKMT function [72,73,355,356]. In order to methylate a certain Lys or Arg residues in a histone, PMTs use a reactive S-adenosyl methionine (SAM) which leaves a methyl group to the respective Lys or Arg residue, becoming S-adenosyl homocysteine (SAH). SAM is used as substrate by proteins other than PMTs and this raises the question whether pharmacological modulation of the SAM binding site may guarantee an adequate selectivity against other SAM-binding proteins. The structural characteristic that differentiates PMTs to other SAM-binding proteins is their elongated active site geometry. In PMTs the SAM binding pocket entrance is in the opposite position of the hydrophobic and narrow histone (Lys or Arg) binding pocket of the methyltransferase; these two tunnels have a contact area where methylation of histones occurs [73,364]. Several reviews published recently, including one of the current journal issue, describe the mechanism of action of PMTs [41,72,73,191,355,357-363]. Because of the importance of PMTs as new biological targets for anticancer therapy, elucidation of their structure is fundamental to undertake drug design campaigns. In the following sections we describe the current structural knowledge available on PMTs. 

### PKMTs 

To date there are 26 crystallized Lys methyltransferases available in the Protein Data Bank (Table **4**). With the exception of DOT1L, all of them share the canonical SET domain [72,73,355, 356] and have S-adenosyl methionine (SAM), S-adenosyl homocysteine (SAH, the product of the methylation reaction), or early inhibitors co-crystallized. Structures with reported inhibitors are DOT1L, EHMT1, EHMT2, SETD7, SMYD1, SMYD2, SMYD3 (Table **4**). Emerging crystallographic structures are likely to allow the implementation of structure-based drug design approaches as these targets become more and more validated for specific anticancer therapies. Moreover, due to the presence of additional proteins interacting with PKMTs during histone methylation, some structures, i.e. MLL1, EHMT1, SETD7, SETD8, SETMAR and SMYD2, were resolved with bound peptide partners. This information is also relevant to the design of potential protein-protein interaction inhibitors. However, to our knowledge, no work has been so far reported in this context. The most direct approach for developing PKMTs ligands seems to be focused on the SET domain. However, whether the most effective approach is to target the SAM or histone binding sites is still subject of investigations, although some co-crystal structures demonstrate that both might be pursued (Table **4**) [73,364]. The complete list of crystallographic structures available PKMTs is reported in Table **3**. PKMTs with resolved SET domain include: MLL1, EHMT1, EHMT2, SUV39H2, NSD1, SETD3, SETD6, SETD7, SETD8, SETD2, SETMAR, ASH1L, SUV420H1, SUV420H2, SMYD1, SMYD2, SMYD3, PRDM1, PRDM4, PRDM10, PRDM11, PRDM12. This structural information may help to predict selectivity of PKMTs ligands to one or more proteins of this family. 

### PRMTs 

As for PKMTs, Arg histone residues can likewise be methylated by protein methyltransferases. Specific PRMTs can mono-methylate or di-methylate, symmetrically or asymmetrically, specific Arg residues in histones through a mechanism similar to the one of PKMTs: a SAM molecule donates a methyl group to an Arg residue becoming SAH [72,73,355]. Interestingly, Arg methylation can be correlated with active transcription or its inhibition. An example is the methylation of Arg 2 of histone 3: when mono-methylated, transcription of DNA is active. Conversely, after di-methylation operated by PRMT6, the transcription is inhibited [402]. PRMTs, like the coactivator-associated arginine methyl-transferase (CARM1) and PRMT5, have been described to play an important role in cancer as their expression increases in breast and prostate cancers, for CARM1, and lymphoma, for PRMT5. Research conducted on new modulating agents of these PRMTs has been documented [72,73]. In particular, CARM1 has been described as a potential oncological target as its interactions with nuclear transcription factors and p53 may represent a new approach for treating cancer [403]. Its crystal structure (Table **5**) has been resolved with a bound indole-based inhibitor, providing new insightful information for the inhibition of this Arg methyltransferase. Other PRMTs that have been crystallized are: PRMT1, PRMT2, PRMT3, ECE2, METTL11A. 

#### DNA Methyltransferases 

DNA methyltransferases are enzymes that methylate DNA patterns involved in several biological functions like gene silencing, X-chromosome inactivation, DNA repair, and reprogramming elements responsible for carcinogenesis [408]. In the last decade, the variety of functions intrinsic to this family of enzymes has propelled research on their biology and pharmacology. In mammals, DNA methylation occurs at the C5 position of cytosine (5mC), predominantly within CpG dinucleotides belonging to the CpG islands. These enzymes use the same substrate of PMTs, a SAM molecule, that is responsible for donating a methyl group to the cytosine nucleotide [359,408-411]. The mechanism of reaction requires the binding of the DNA methyltransferase to the DNA strand. This interaction projects the double helix outwards, thereby causing a cytosine base-flipping. A subsequent attack from the conserved nucleophile cysteine on the cytosine C6 is followed by the transfer of the methyl group from SAM to the activated cytosine C5 [408,410]. 

DNMTs can be divided into three groups according to their function: DNMT1, the most abundant DNA methyltransferase, regarded as a maintenance enzyme; DNMT3s A and B considered *de novo* methyltransferases because they have the ability to newly methylate cytosines; DNMT3L itself (part of the second group of DNMTs) does not have any catalytic activity but it is required for the function of DNMT3A and B; finally, DNMT2, the least studied DNA methyltransferase, has been solved by X-ray crystallography and biochemical data demonstrate that it functions as an aspartic acid transfer RNA (tRNAAsp). Recent data suggest that there are additional functions for this DNA methyltransferase [227,412]. The structure of DNMTs is mainly composed of a large N-terminal region, with several domains and variable size, and a C-terminal domain. While the N-terminal domain has several distinct regulatory functions, the catalytic site is located in the C-terminal domain [408,411]. Among the regulatory functions of the N-terminal region, there are the guidance of these proteins towards the nucleus and their interaction with DNA and chromatin. The C-terminal domain is more conserved between bacterial and eukaryotic DNMTs and, in its active site, a set of ten residues constitutes the motif for all DNMTs that methylate C5 cytosines. The core of the catalytic domain of all DNMTs is common along this enzyme family and is termed *AdoMet-dependent MTase fold*. In this domain, conserved regions are involved in catalysis and co-factor binding, whereas the non-conserved region is involved in DNA recognition and specificity to methylate certain cytosines [408,411]. The ensemble of structural data of DNMTs is show in Table **6**. 

It is important to note that the close relationship between DNMTs functions on the cell and cancerogenesis led this family of proteins to be intensively studied for a number of cancer pathologies (see previous chapters). Comprehensive reviews providing more detail on inhibitor development and major milestones in targeting DNMTs have been published recently [65,227,357,360, 361,413]. 

#### Demethylases 

The focus in the last decade in understanding protein methylation led to the discovery of histone demethylases. The existence of this protein family was first described by Shi et al., who identified the first protein with histone demethylase activity, the lysine-specific demethylase 1 (LSD1) [421]. Since then, histone demethylation was identified as an important regulator for gene transcription and the interest in this protein family increased rapidly in subsequent years. Tsukada *et al.* [422] described a member of the JMJC (Jumonji C) domain family of proteins as having demethylase activity. Soon thereafter, 30 members of the JMJC domain family were found employing bioinformatics approaches, but only 18 of them have been reported to exhibit demethylase activity [423]. Histone demethylases are currently divided into two families: LSD demethylases and JMJC demethylases. These two protein families differ in their mechanism of Lys demethylation, in their structure and in substrate specificity. 

The LSD family has two members, LSD1 and LSD2, and uses FAD to demethylate the histone Lys residues H3K4 and H3K9 through a FAD-dependent oxidative reaction [424]. Through this mechanism, both demethylases are only able to operate on mono- or-di-methylated Lys residues. Currently, structural data is only available for LSD1 (Table **7**). The complete structure of the LSD1 comprises an amine oxidase domain with two lobes: a FAD binding region and the substrate binding region. The latter is responsible for the enzymatic activity of the LSD1, as the active site is located at the interface of the two lobes and is similar to conventional FAD-dependent amine oxidases [424]. Furthermore, this protein has an N-terminal SWIRM (derived from Swi3p, Rsc8p, and Moira) domain which is responsible for protein-chromatin interactions. The SWIRM and amino oxidase domains are packed together, forming a globular structure. Interestingly, LSD1 was demonstrated to demethylate *in vitro* methylated peptides but is itself unable to demethylate methyl-Lys of the nucleosome [424-426]. Only in complex with the co-repressor protein (CoREST) this protein is able to demethylate nucleosomes, indicating that LSD1 protein partners are likely to be involved in enzymatic activity *in vivo* [424]. 

All demethylases of the JMJC family have a JMJC domain in common which has been demonstrated to fold into eight β-sheets, in a jellyroll-like β-fold [423,424,427,428]. In the inner part of this jellyroll structural motif, the active site is buried and has a Fe^2+^ metal which is coordinated by α-ketoglutarate (α-KG) and three conserved residues, a Glu and two His. The enzyme uses molecular oxygen in order to convert the methyl group of the methylated Lys in hydroxymethyl, which is successively released as formaldehyde. This type of active site permits JMJC demethylases to demethylate mono- and di-methylated lysines, and to also act on tri-methylated lysines [423,424,427-429]. The jellyroll motif is surrounded by other structural elements which help to maintain the structural integrity of the catalytic core and contribute to substrate recognition [424]. 

LSD and JMJC demethylases have been reported as regulators of various cellular processes. A considerable effort is currently directed to the discovery of small-molecule inhibitors able to modulate their catalytic activity. The number of structures of demethylases available from the PDB is growing rapidly (Table **7**) [430,431]. 

### Ubiquitylation and Sumoylation 

The formation of an isopeptide bond between the C-terminal Gly76 of ubiquitin (Ub) and an ε-amino group on one of the internal Lys residues of a substrate protein is known as ubiquitylation. This PTM of proteins occurs through a series of enzymatic steps involving E1, E2 and E3 proteins. Firstly, Ub is activated to form a thioester with a specific cysteine residue located in the E1 enzyme, also known as ubiquitin-activating (UBA) enzymes. The activated Ub is subsequently transferred to one of the Ub-conjugating enzymes (E2) and, eventually, an Ub ligase (E3) interacts with the ubiquitylation target and transfers the activated Ub from E2 to one of the Lys on the protein substrate, including histones [12]. In contrast to other histone PTMs, ubiquitylation involves a significant change at molecular level since the Ub is a 76 amino acids protein that marks proteins for ATP-dependent proteolytic degradation by 26S proteasomes in the so called Ubiquitin-proteasome system (UPS). Some E1, E2 and E3 enzymes have been found to be responsible for the addition and removal (*via* DUB enzymes) of ubiquitin from histones H2A and H2B [15,24]. These studies highlighted that H2A and H2B ubiquitylation, especially the mono-ubiquitylation, plays a key role in regulating several epigenetic processes within the nucleus, including transcription initiation, elongation, silencing and also DNA repair [15,468-470]. A correlated PTM is the sumoylation, which consists in the attachment of ubiquitin-like fragments on histone Lys residues through ubiquitylating enzymes [471]. This PTM is still scarcely characterized but appears to exert a transcriptional repression role by competing with ubiquitylation at the substrate level [472]. Some three-dimensional structures of UBAs have been resolved [473,474], though the current knowledge on the ubiquitylating enzyme cascade is still fragmentary in relation to the involvement of these proteins as epigenetic controllers of histones. Consequently, the road to the full comprehension of structural data and their involvement in the mechanistic processes is still a major area of research. Moreover, the identification of small-molecule modulators of ubiquiting ligases is currently an active field of research for novel anti-cancer drugs [475-479]. Small molecules have been described for Mdm2 and R7112, but their action seems to primarily affect the ubiquitination mechanism of p53 and not those mechanisms leading to the modification of histone tails [479]. Further insights on the structural and functional roles of ubiquitylating enzymes are needed as they are expected to let emerge new biological targets for anticancer therapies [478]. 

### ADP-ribosylation 

Histone proteins have been described to be mono- and poly-ADP-ribosylated, thus these PTMs have been directly linked to the epigenetic code [480]. The transfer of one ADP-ribose from NAD^+^ to specific residues is known as mono-ADP-ribosylation and is catalyzed by ADP-ribosyltransferases referred to as ARTC (Clostridia-toxin-like) or ARTD (diphtheria toxin-like; formerly known as PARPs), as well as by mitochondrial SIRT4 and nuclear SIRT6 sirtuin family members [480,481]. The subcellular location of ARTC does not allow mono-ADP-ribosylation of histone tails as these proteins are ecto-enzymes [480]; *vice versa* some ARTD members and SIRT6 can be involved in nuclear mono-ADP-ribosylation of histones. Furthermore, ADP-ribosylation of protein-linked ADP-ribose results in poly-ADP-ribosylated proteins, a reaction that is catalyzed by certain members of the ARTD family. ARTD1 (also known as PARP1) activity causes chromatin decondensation by poly-ADP-ribosylating core histones and the linker histone H1 [482]. The full understanding of histone ADP-ribosylation is currently a major topic of research, especially for the identification of ADP-ribosylation sites *in vivo* and the development of specific tools to locate these histone modifications [483,484]. Several studies indicate that histones are covalently modified by mono-ADP-ribose in response to genotoxic stress, and others that the extent of mono-ADP-ribosylation of histones depends on the cell cycle stage, proliferation activity and degree of terminal differentiation [485-490]. 

While the role of ADP-ribosylation as histone PTM is being elucidated [491-495], it should be acknowledged that ARTDs and sirtuins emerged in the last decades as important biological targets for many other cellular processes [484]; moreover several medicinal chemistry approaches aimed at the discovery of novel inhibitors recently appeared [483,490,496-500]. The most studied ADP-ribosylating enzyme has been ARTD1, however growing evidences indicate important roles of other mono- and poly-ADP-ribosylating enzymes, including tankyrases [490,501-503]. Crystallographic and NMR data exist for some ARTD members and have been recently described [502]. ARTCs are less characterized from a structural point of view. A review on this topic is available in the current journal issue [482]. For available structural data on ADP-ribosylating sirtuins see the previous sections. 

Even though the role that ADP-ribosylation plays in histone modifications has not yet been completely characterized, it should be acknowledged that this PTM was shown to largely contributes to the epigenetic control of several important process, such as regulation of genomic methylation patterns in gene expression [504,505], effects on chromatin structure [506-508] and transcriptional activator and co-activator functions [492]. It is expected that further definition of specific functions of ADP-ribose modifications will incite efforts towards the identification of new therapeutic routes based on small-molecule inhibitors of these enzymes. 

### Phosphorylation 

Histone phosphorylation plays a key role in cell cycle control, DNA repair, apoptosis, gene silencing, chromatin structure and cellular differentiation [8,11,12,509-515]. It occurs on Ser, Thr, Tyr and His residues and is not limited to histone tails [12,516-518]. The regulation of histone phosphorylation is operated by the enzymatic activity of kinases that transfer a phosphate group to a target residue and phosphatases that counter this activity by hydrolyzing phosphates. Identified kinases that contribute to dynamic phosphorylation marks on histones include Aurora B [511], MSK1 [513], HHK [517], among others [514,519]. Structural data about some of these kinases are documented in the available literature while other kinases are still not well described. The mechanism and pharmacological interventions on histone phosphorylation are still poorly understood. However, it is worth noting that the pattern of expression of histone H4 His kinase (HHK) has been suggested as a useful diagnostic marker for hepatocellular carcinoma [517]. To our knowledge, no compounds addressing the pharmacological modulation of histone phosphorylation have been approved to date, though several Aurora kinase inhibitors have been identified and some have entered phase II clinical trials [24]. 

### Glycosylation 

The addition of N-acetylglucosamine (GlcNAc) to Ser and Thr residues (O-GlcNAc) of nuclear and cytoplasmic proteins is an unconventional type of glycosylation that represents an important PTM. This kind of glycosylation is atypical for at least three reasons. First, it involves the addition of a single monosaccharide; second, it takes place in the cytoplasm and modified glycoproteins are usually nuclear and cytoplasmic, including RNA polymerase II, ER, c-Myc proto-oncogene and histones; third, it is reversible in the way that the monosaccharide can repeatedly be attached and detached. In general, the addition of O-GlcNAc is reciprocal with Ser and Thr phosphorylation, either by modification of the same residue or nearby residues [520]. This PTM modification is regulated by only two enzymes: a glycosyltransferase that catalyzes the transfer of GlcNAc to substrate proteins, also known as O-GlcNAc transferase (OGT), and a glycoside hydrolase, also known as O-GlcNAcase (OGA) or O-N-acetylglucosaminidase, that catalyzes the hydrolysis of the glycosidic linkage [521]. While it is interesting to note that in mammalians only these two highly conserved enzymes are responsible of O-GlcNAc cycling, it is worth emphasizing that the targeting of these enzymes is highly specific and is controlled by many interacting subunits. 

Only recently O-GlcNAc was linked to the epigenetic code, demonstrating that the four core histones are substrates for O-GlcNAc modifications and cycle genetically and physically in order to interact with other PTMs of histones [520,522-526]. In addition, OGT has been described to target key members of the Polycomb and Trithorax groups [527]. The role of O-GlcNAc as a PTM able to alter key cellular signaling pathways has been discussed by linking epigenetic changes and metabolism [523]. Even though the role of ‘conventional’ glycosylation in cancer [528-532] and in ageing [533] is well recognized, little is known on the role of O-GlcNAc histone modification in cancer [531]. 

Details about how OGT recognizes and glycosylates its protein substrates, including histone proteins, were almost unknown until recent years, when novel protein structural data became available (PDB codes: 1W3B and 3TAX) [521,534,535]. In 2011, the first two crystal structures of human OGT were solved: a binary complex with uridine 5'-diphosphate (UDP) and a ternary complex with UDP and a peptide substrate, including the catalytic region (PDB codes: 3PE3 and 3PE4) [536]. Additional reports discussing the mechanism underlining OGT and OGA activities as well as small-molecule inhibitors and drug discovery methodologies have recently appeared [537-543]. Glycosyltransferases have been recently used to derive glycosylated analogues of novobiocin with improved activity against several cancer cell lines [544]. While glycosylation represents an emerging PTM of histones that is expected to provide new biological clues in cancer epigenetics, both OGT and OGA have not yet been validated for drug design purposes. Nevertheless, small inhibitors for both enzymes, e.g. by PUGNAc and related derivatives, have been described in literature [521,545-548]. It is expected that the discovery of other inhibitors of these enzymes, for use as cellular probes, will help the full understanding of O-GlcNAc as a covalent histone modification and will contribute to foster research toward new epigenetic therapeutic agents. 

### Carbonylation 

Covalent modification of cysteines by reactive carbonyl species (RCS) is known as carbonylation. Production of RCS is a feature of redox signaling by enzymes like peroxiredoxins, tyrosine phosphatases/kinases and transcription factors (e.g. p53, NFkB and Nrf2) [549,550]. Intracellular levels of RCS are originated from non-enzymatic and enzymatic peroxidation of lipids, especially arachidonic acid; this process generates unsaturated aldehydes (enals), like 4-hydroxy-2-nonenal (4HNE), crotonaldehyde and acrolein, as well as unsaturated ketones (enone), like cyclopentenone prostaglandins. RCSs are able to act on membrane and cytosolic proteins, while little is known about the actions of RCS on nuclear proteins and the overall extent of changes in cell signaling and gene expression. Histones have been found to undergo carbonylation [549-551]. However, differently to other PTMs, carbonylation occurs without the specific action of enzymes as RCSs are directly responsible for the chemical attack of histone modification sites. In addition, the absence of enzymes that oppose histones carbonylation seems to predispose carbonylated histones to accumulate. This fact was observed in rat pheochromocytoma cells following alkylating stress [550]. 

Because carbonylation is in general a hallmark of protein oxidation it has been mostly connected to aging, inflammaging, caloric restriction and age-related pathologies [552]. There is scarse knowledge about how carbonylation enzymes might govern other cellular redox processes, including those leading to cancer *via* histone covalent modifications. 

### Citrullination/Deimination 

Citrullination or deimination is exerted by protein-arginine deiminases (PAD), in particular PADI4 [553], and serve as a sort of Arg demethylase as it converts methyl-arginines to citrulline with release of methylamine, thereby regulating histone Arg methylation [12,554,555]. This kind of mechanism is not to be considered a demethylation reaction in a strict sense as it produces a citrulline instead of a charged Arg residue. Citrullination of histones has been described in relation to its capacity to antagonize arginine methylation by CARM1 [554], but its role in cancer diseases is starting to be delineated now [556,557]. 

Some classes of compounds have been described to inhibit PADI4 [558-561]. There are several available crystallographic structures of these enzymes, also in complex with inhibitors (PDB codes: 1WD8, 1WD9, 1WDA, 2DEW, 2DEX, 2DEY, 2DW5, 3APM, 3APN, 3B1T, 3B1U and 4DKT) [560-563]. It is expected that further work in this direction will help to elucidate the role of histone citrullination in cancer diseases. 

### Biotinylation 

Biotinylation is the attachment of biotin to a protein, nucleic acid or other molecule. Biotinylation of histones was described in several histone variants and is likely to be involved in gene silencing, cell proliferation, and cellular response to DNA damage [8,564-569]. Amino-acid residues that undergo biotinylation have been identified in some recent studies [570,571] but the role of histone biotinylation in cancer pathologies remains largely unclear. Recent findings suggest that an altered biotin status in some population subgroups might affect chromosomal stability and cancer risk [569]. This PTM is catalyzed by biotin-protein ligase (also known as holocarboxylase synthetase) which specifically acts on substrate proteins by attaching biotin covalently. The opposing catalytic activity is exerted by biotinidase. In both cases, no structural data of the human proteins is yet available. Nonetheless, some structures of biotin-protein ligase of *Pyrococcus horikoshii *have been recently published (PDB codes: 2DXU, 2DZC, 2DXU, 1WQW) [572,573]. To the best of our knowledge no drug- or lead-like ligand has been yet identified to interfere with histone biotinylation. 

### Other PTMs 

Other PTMs have been reported in the literature such as histone tail clipping and histone proline isomerization [12]. Histone tail clipping consists in the removal of the N-terminal tail of a histone with potential consequences for transcription and many other events involving chromatin remodeling. The capacity to clip histone tails was demonstrated to date in yeast H3 [574]. Proline isomerization is a particular PTM that does not imply a covalent modification of histones but a *cis*-*trans* isomerization of Pro residues. As only Pro amides allow this conformational flexibility, proline isomerization is considered to play important biochemical roles including control of protein folding, initiation of transmembrane signaling, recognition of peptide antigens and regulation of peptide breakdown. In the context of histone modifications, Pro isomerization was found to be catalyzed by Pro isomerase Fpr4 in *Saccharomyces cerevisiae.* Regulating transcription and cross-talk with histone Lys methylation was also described for Fpr4 [575]. 

The catalytic effectors of these PTMs are subject of investigations [12], while proteomics techniques aimed at elucidating their biological roles are under development [8]. Further tools and studies are expected to provide new insights into the mechanism, dynamics and impact of these modifications in association with cancer pathologies. 

### miRNAs Regulating Proteins 

Noncoding miRNAs are capable of inducing heritable changes in gene expression profiles without altering the DNA sequence. miRNAs and Piwi-interacting (P- element-induced wimpy testes) RNAs (piRNAs) are classes of small RNAs that are generated by the activity of RNaseIII enzymes; they have a variety of biological functions, such as heterochromatin formation, mRNA inactivation and transcriptional regulation. Their role contributes to global epigenetic mechanisms as miRNAs can: i) modulate the expression of chromatin remodelers involved in epigenetic modifications (e.g. HDACs, DNMTs and Polycomb proteins) with specific *epi-miRNAs* [576]; ii) guide the recruitment of chromatin remodelers on DNA either by the interaction with promoter-associated RNA (pRNA) or by directly targeting complementary promoter sequences, thus promoting transcriptional gene silencing [577,578]; iii) be subjected to epigenetic modifications of their corresponding promoter loci [579]. 

As seen in the previous chapter, deregulation of miRNAs is associated with the development and progression of several cancer types [61,96,576,580-583]. The bioactivity of miRNA is generally linked to Argonaute (Ago)-family proteins that serve as a direct interaction partner of the miRNA within the RNA-induced silencing complex (RISC). In particular, the miRNA guides the RISC to its target mRNA, while Ago protein complex leads to silencing of gene expression by repressing mRNA translation or by inducing deadenylation-dependent mRNA decay [22,584-586]. 

The research aimed at elucidating structure and function of miRNA-pathway components has been stimulated in the last year by crystallographic data and NMR spectra of several proteins that have been reviewed in another article of this journal issue [61]. It should be noted that, since the biogenesis and function of microRNAs and endo- and exo- siRNAs are regulated by Ago2, the identification of non- miRNA compounds, that can be used to block the cycle of miRNA loading, might constitute a new therapeutic approach to several cancer diseases. The design of new modulators for the miRNA/siRNA pathway might be facilitated by the development of new assay for HTS, while the discovery of new bioactive compounds would have the potential to broad their applications in functional studies of Argonaute and individual miRNAs in cell biology and human disease [587]. It is therefore expected that future studies in epigenetic regulation of miRNA expression coupled to downstream signaling pathways will most likely lead to the discovery of novel drug targets for novel anticancer therapies [61,96,588]. 

## 4 SUMMARY AND DRUG DISCOVERY PERSPECTIVES 

Major research efforts are currently directed toward the discovery of new small-molecules able to modulate target proteins described in the previous chapters which are involved in chromatin remodeling and DNA methylation [19,231]. Recent success stories document the potential to successfully interfere with the epigenetic code with small organic molecules [25]. Moreover, the first pre-clinical and clinical results obtained in the last years, especially for HDACs and DNMTs, lead to the perception that many other *epidrugs* might be effective as combination therapies to control the process of genesis and progression of several forms of cancer. While it is hard to predict whether these results will eventually guide to novel anticancer therapies, several aspects concerning epigenetic drug design still need to be fully assessed. Here as follows we describe some of the requirements, challenges and perspectives. 

### Validation of Anti-cancer Targets 

Despite the numerous studies in the field, much of the research still focus on elucidating the biological functions of the majority of the above-discussed epigenetic enzymes. Beyond the problem of the identification of specific drug-like compounds, the most relevant challenge remains to establish the biological extent by which a putative pharmacological action would impact specific signaling pathways or specific cancer pathologies. This fact is tightly related to the proper validation of the target, a crucial step in the drug discovery pipeline [589]. In this direction, several aspects still need to be addressed. For instance, the discovery of new biomolecules to use as cellular probes, the design of bioassays to measure biological activities and the possibility to setup and perform HTS. At present, most of the enzymes described have not been fully validated (Fig. **1**) [589]. It is worth emphasizing that the increasing availability of structural data, herein described, should be relevant for the identification of new *tool compounds*. These could boost the biological research to validate and determine the specific functions of epigenetic targets in cancer diseases. 

### Understanding the Selectivity/Polypharmacology 

It is now clear that a target-centric approach consisting in the design of small-molecules having maximal selectivity profiles, also referred to *magic bullets* [590], has been very successful for certain diseases but failed in other cases [591]. On the other hand, the intrinsic polypharmacological nature of many chemical scaffolds might result in lack of selectivity on epigenetic targets as several families use common substrates and cofactors (e.g NAD^+^/NADH, FAD, SAM, AcCoA, α-Ketoglutarate and ATP) to exert their catalytic activity. For these reasons, extensive assessments of small-molecules need to be performed in order to study their impact on the epigenome. Collecting comprehensive activity profiles is of particular importance as sought compounds might be selective or promiscuous depending on the biological application [592]. Of particular interest is the elucidation of the polypharmacological behavior of dietary and nutraceutical components. Indeed, many clinical, physiopathological and epidemiological studies highlighted the detrimental or beneficial role of nutritional factors in conjunction to epigenetic alterations [27,37,66,91,238,593]. 

### Mechanism of Action 

**Fig. (2):**
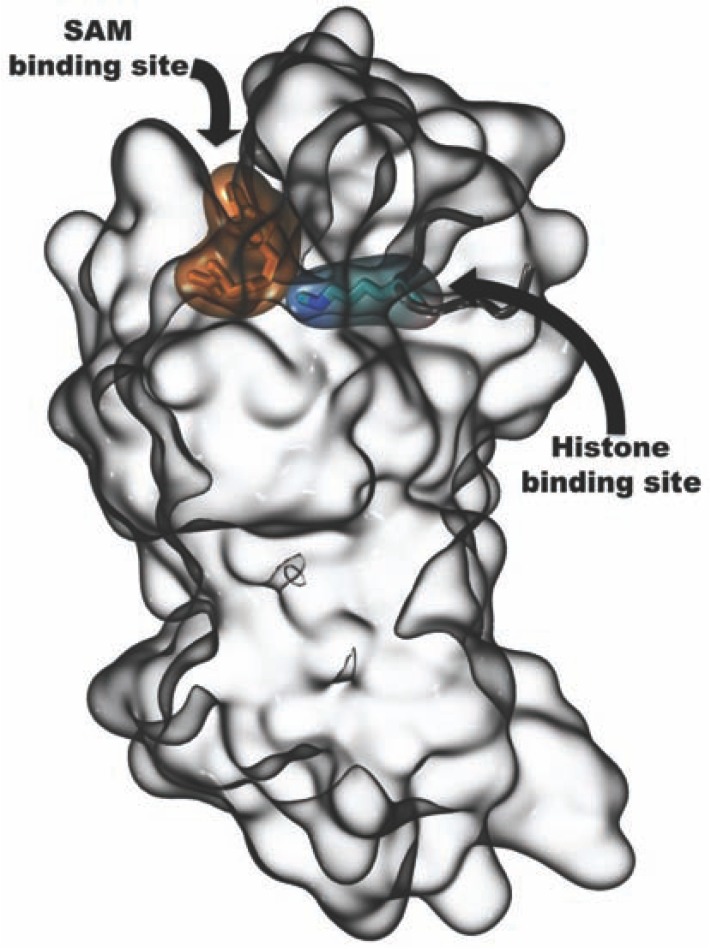
Depiction of SAM and histone binding sites of SETD8 methyltranferase. Assessing the mode of action implies a full understanding of the more appropriate mechanism of inhibition operated by small-molecules.

A deeper understanding of the mechanisms of action of small-molecules needs to be obtained. These mechanisms include: allosteric regulation, inhibition/activation and enzymatic kinetics (e.g. reversible/irreversible, substrate and cofactors competition/non-competition). These tasks may be challenging, especially when natural and/or dietary bioactive components are involved. A representative example is the current diatribe concerning resveratrol and its analog compounds as modulators of sirtuins [271,594-597]. Besides, the study of the action mechanisms plays a critical role also during the early-stage development of novel bioactive compounds. For instance, in the case of methyltransferases a question is raised on whether drug design approaches should be addressed to target the S-adenosyl methionine cofactor binding site or the substrate binding site where Lys or Arg residues of the histones are methylated (Fig. **2**). 

### Protein Flexibility and Protein-protein Interaction Mechanisms 

A number of multiprotein complexes govern catalytic mechanisms associated to epigenetic enzymes. Knowing how these molecular machineries are constituted, how their dynamic behavior influences the catalytic activities, and how this behavior ultimately influences the processes of tumorigenesis and cancer progression is of extreme importance. For example, the methyltransferase EZH2, which is part of the core of the Polycomb repressive complex (PRC2), was recently subject of crystallographic studies indicating protein-protein interaction patterns within the PRC2 [598]. Similarly, in many cases, a low amount of information is available, at a molecular level, about the flexibility and the conformational ability of epigenetic enzymes to recognize and interact with histone substrates. It becomes an even more complicated task when additional components, like co-repressors, are required (e.g. in LSD1). In this direction molecular modeling techniques promise to be a helpful tool to explore these molecular mechanisms. 

### Application of Computer-aided Drug Design Techniques 

In the last decades computer-aided drug design (CADD) techniques have been successfully used to guide the selection of new compounds with predefined biological activity. These techniques include a variety of chemoinformatic and computational chemistry tools. In particular, virtual screening procedures are well established for a rapid and cost-effective evaluation of large chemical libraries of commercial compounds [599-601]. The growing availability of three-dimensional structures presented in the previous chapter raises the possibility to deploy structure-based drug design (SBDD) techniques, like docking or pharmacophore screenings, in search of novel compounds able to modulate these targets [51,601,602]. Several extensive reviews have been published recently on this topic [19,227,231,361,603]. 

### Use of New and Successful Paradigms of Medicinal Chemistry 

The long-term effectiveness of traditional medicinal chemistry approaches has not yet been fully demonstrated to the development of new *epidrugs*. On the other hand, new medicinal chemistry paradigms such as the repurposing of known drugs [604,605] and the screening of nutraceutical components and natural compounds [606,607], are attracting a lot of interest in different areas of research and could also be effective for targeting epigenetic enzymes. 

Apart from new paradigms of medicinal chemistry, it should also be noted how, established but powerful drug design methodologies, are currently being re-evaluated. An example is the resurgence of phenotypic screening, i.e. where compounds are screened in cellular or animal disease models to identify those causing desirable changes in the phenotype. These kind of screens accounted in the last decade for a surprising number of identification of block-buster drugs with novel mechanisms of action in respect to target-based screening [608]. Since the assessment of the tightly-regulated mechanisms of epigenetic modifications might be difficult to track-down in all cases, the use of phenotypic screens might constitute a valuable tool for the identification of new *epi*-compounds [609]. 

Remaining all the above-mentioned considerations, it is unquestionable that epigenetics framework will play a major role in the near future to develop new therapies against cancer. We hope that this review will stimulate new and original initiatives in this direction. 

## Figures and Tables

**Table 1.** Available 3D Structure of Human and Bacterial HDACs

Class	Name	Organism	PDB ID	Ligand	Domain	Reference
**I**	**HDAC2**	*Homo sapiens*	3MAX	N-(4-aminobiphenyl-3-yl)benzamide	Catalytic Domain	[247]
	**HDAC3**	*Homo sapiens*	4A69	Nuclear receptor corepressor 2 and inositol tetraphosphate	Catalytic Domain	[248]
	**HDAC8**	*Homo sapiens*	1T64	Trichostatin A	Catalytic Domain	[249]
			1T67	M344 (B3N^a^)	Catalytic Domain	
			1T69	SAHA	Catalytic Domain	
			1VKG	Cra-19156	Catalytic Domain	
			1W22	Hydroxamic acid inhibitor	Catalytic Domain	[250]
			2V5W	Acetylated substrate	Catalytic Domain (mutate)	[251]
			2V5X	Hydroxamic acid inhibitor	Catalytic Domain	
			3EW8	M344 (B3N^a^)	Catalytic Domain (mutate)	[252]
			3EWF	Substrate Peptide	Catalytic Domain (mutate)	
			3EZP	M344 (B3N^a^)	Catalytic Domain (mutate)	
			3EZT	M344 (B3N^a^)	Catalytic Domain (mutate)	
			3F06	M344 (B3N^a^)	Catalytic Domain (mutate)	
			3F07	APHA	Catalytic Domain	
			3F0R	Trichostatin A	Catalytic Domain	
			3MZ3	M344 (B3N^a^)	Catalytic Domain (Co2+)	[244]
			3MZ4	M344 (B3N^a^)	Catalytic Domain (Mn2+)	
			3MZ6	M344 (B3N^a^)	Catalytic Domain (Fe2+)	
			3MZ7	M344 (B3N^a^)	Catalytic Domain (Co2+)	
			3RQD	Largazole	Catalytic Domain	[253]
			3SFF	Aminoacid derived inhibitor	Catalytic Domain	[254]
			3SFH	Aminoacid derived inhibitor	Catalytic Domain	
**IIa**	**HDAC4**	*Homo sapiens*	2H8N		Glutamine Rich Domain	[255]
			2O94		Glutamine Rich Domain	
			2VQJ	Trifluoromethylketone inhibitor	Catalytic Domain	[256]
			2VQM	Hydroxamic acid inhibitor	Catalytic Domain	
			2VQO	Trifluoromethylketone inhibitor	Catalytic Domain (mutate)	
			2VQQ	Trifluoromethylketone inhibitor	Catalytic Domain (mutate)	
			2VQV	Hydroxamic acid inhibitor	Catalytic Domain (mutate)	
			2VQW		Catalytic Domain (mutate)	
	**HDAC7**	*Homo sapiens*	3C0Y		Catalytic Domain	[257]
			3C0Z	SAHA	Catalytic Domain	
			3C10	Trichostatin A	Catalytic Domain	
**IIb**	**HDAC6**	*Homo sapiens*	3C5K		Zinc Finger Domain	
			3GV4	Ubiquitin C-terminal peptide	Zinc Finger Domain	
			3PHD	Ubiquitin	Zinc Finger Domain	[258]
**Bacterial**	**HDAH**	*Alcaligenes sp.*	1ZZ0	Acetate	Catalytic Domain	[259]
			1ZZ1	SAHA	Catalytic Domain	
			1ZZ3	CypX	Catalytic Domain	
			2GH6	Trifluoromethylketone inhibitor	Catalytic Domain	[260]
			2VCG	ST-17	Catalytic Domain	[261]
	**HDLP**	*Aquifex Aeolicus*	1C3P		Catalytic Domain	[239]
			1C3R	Trichostatin A	Catalytic Domain	
			1C3S	SAHA	Catalytic Domain	
	**APAH**	*Mycoplana ramosa*	3Q9B	M344	Catalytic Domain	[262]
			3Q9C	N8-acetylspermidine	Catalytic Domain (mutate)	
			3Q9E	Acetylspermine	Catalytic Domain (mutate)	
			3Q9F	CAPS	Catalytic Domain	
		*Burkholderia pseudomallei*	3MEN		Catalytic Domain	[263]

a PDB ligand ID

**Table 2.** Available Three-dimensional Structures of Human and Bacterial Sirtuins

Name	Organism	PDB ID	Ligand	Reference
**SIRT2**	*Homo sapiens*	1J8F	-	[272]
**SIRT3**	*Homo sapiens*	3GLR	Acetyl-lysine AceCS2 peptide	[273]
		3GLS	-	
		3GLT	Thioacetyl-lysine AceCS2 peptide	
		3GLU	AceCS2 peptide	
**SIRT5**	*Homo sapiens*	2B4Y	ADPR	[270]
		2NYR	Suramin	
		3RIG	-	[274]
		3RIY	NAD^+^	
**SIRT6**	*Homo sapiens*	3K35	ADPR	[275]
		3PKI	ADPR	
		3PKJ	2'-N-Acetyl-ADPR	
**CobB (Sir2)**	*Thermotoga maritima*	1YC5	Nicotinamide	[268]
		2H2D	p53 peptide	[276]
		2H2F	p53 peptide	
		2H2G	Histone H3 peptide	
		2H2H	Histone H4 peptide	
		2H2I	-	
		2H4F	p53 peptide/NAD^+^	[277]
		2H4H	p53 peptide/NAD^+^	
		2H4J	p53 peptide/Nicotinamide/2-O-acetyl-ADPR	
		2H59	p53 peptide/ADPR	
		3D4B	p53 peptide/DADMe-NAD^+^	[278]
		3D81	S-alkylamidate intermediate	
		3JR3	Acetylated Peptide	[279]
		3PDH	Propionylated p53 peptide	[280]
**CobB2 (Sir2Af2)**	*Archaeoglobus fulgidus*	1YC2	NAD/ADPR/Nicotinamide	[268]
		1S7G	ADPR/NAD	[281]
		1MA3	Acetylated p53 peptide	[282]
**HST2**	*Saccharomyces cerevisiae*	1Q14	-	[283]
		1Q17	ADPR	[284]
		1Q1A	Histone H4 peptide/2-O-acetyl-ADPR	
		1SZC	Histone H4 peptide/CarbaNAD	[285]
		1SZD	Histone H4 peptide/ADPR	
		2OD2	Acetylated Histone H4 peptide/CarbaNAD	[269]
		2OD7	Acetylated Histone H4 peptide/ADP-HPD	
		2OD9	Histone H4 peptide/ ADP-HPD/Nicotinamide	
		2QQF	Acetylated Histone H4 peptide/ADP-HPD	
		2QQG	Histone H4 peptide/ ADP-HPD/Nicotinamide	
**CobB1**	*Archaeoglobus fulgidus*	1ICI	NAD^+^	[267]
		1M2G	ADPR	[286]
		1M2H	ADPR	
		1M2J	ADPR	
		1M2K	ADPR	
		1M2N	2-O-acetyl-ADPR	
**CobB**	*Escherichia coli*	1S5P	Acetylated Histone H4 peptide	[286]
**Sir2**	*Saccharomyces cerevisiae*	2HJH	Acetyl-ribosyl-ADP/Nicotinamide	
**Sir2**	*Plasmodium falciparum*	3JWP	AMP	
		3U31	histone 3 myristoyl lysine 9 peptide/ NAD^+^	[287]
		3U3D	histone 3 myristoyl lysine 9 peptide	

**Table 3.** Available Three-dimensional Structures of Human and Bacterial Human and Bacterial HATs

Class	Family	Name	Organism	PDB ID	Ligand	Domain	Reference
**Type A**	**GNAT**	**KAT2A (GCN5)**	*Homo sapiens*	1F68		Bromodomain	[292]
				1Z4R		HAT Domain	[293]
				3D7C		Bromodomain	[294]
			*Saccharomyces cerevisiae*	1E6I		Bromodomain	[295]
				1YGH		HAT Domain	[296]
		**KAT2B (PCAF)**	*Homo sapiens*	1CM0		HAT Domain	[297]
				1JM4		Bromodomain	[298]
				1N72		Bromodomain	[299]
				1WUG	NP1	Bromodomain	[300]
				1WUM	NP2	Bromodomain	
				1ZS5	MIB	Bromodomain	
				2RNW		Bromodomain	[301]
				2RNX		Bromodomain	
				3GG3		Bromodomain	[294]
		**HPA2**	*Saccharomyces cerevisiae*	1QSM	AcCoA	HAT Domain	[302]
				1QSO		HAT Domain	
	**p300/ CBP**	**p300**	*Homo sapiens*	1L3E		CH1 domain	[303]
				1P4Q		CH1 domain	[304]
				2K8F		Taz2 Domain	[305]
				3BIY	Lys-CoA	HAT Domain	[306]
				3I3J		Bromodomain	[294]
				3IO2		Taz2 Domain	[307]
				3P57		Taz2 Domain	[308]
		**CBP**	*Homo sapiens*	1JSP		Bromodomain	[309]
				1LIQ		CH1 domain	[310]
				1WO3		CHANCE Domain (mutate)	[311]
				1WO4		CHANCE Domain (mutate)	
				1WO5		CHANCE Domain (mutate)	
				1WO6		CHANCE Domain (mutate)	
				1WO7		CHANCE Domain (mutate)	
				1ZOQ		IRF-3 Binding Domain	[312]
				2D82	TTR	Bromodomain	[313]
				2KJE		Taz2 Domain	[314]
				2KWF		KIX Domain	
				2L84	J28	Bromodomain	[315]
				2L85	L85	Bromodomain	
				2RNY		Bromodomain	[301]
				3DWY		Bromodomain	[294]
				3P1C		Bromodomain	
				3P1D		Bromodomain	
				3P1E	DMSO	Bromodomain	
				3P1F	3PF	Bromodomain	
				3SVH	KRG	Bromodomain	
				4A9K	Tylenol	Bromodomain	[316]
			*Mus musculus*	1F81		Taz2 Domain	[317]
				1JJS		IRF-3 Binding Domain	[318]
				1KBH		IRF-3 Binding Domain	[319]
				1KDX		KIX Domain	[320]
				1L8C		Taz1 Domain	[321]
				1R8U		Taz1 Domain	[322]
				1SB0		KIX Domain	[323]
				1TOT		ZZ Domain	[324]
				1U2N		Taz1 Domain	[325]
				2AGH		KIX Domain	[326]
				2C52		SRC1 Interaction Domain	[327]
				2KA4		Taz1 Domain	[328]
				2KA6		Taz2 Domain	
				2KKJ		Nuclear Coactivator Binding Domain	[329]
				2L14		Nuclear Coactivator Binding Domain	[330]
	**MYST**	**KAT5 (TIP60)**	*Homo sapiens*	2EKO		Histone tail binding domain	
				2OU2	AcCoA	HAT Domain	
		**KAT6A (MOZ)**	*Homo sapiens*	1M36		Zinc Finger Domain	
				2OZU	AcCoA	HAT Domain	
				2RC4	AcCoA	HAT Domain	[331]
		**KAT8 (MOF)**	*Homo sapiens*	2GIV	AcCoA	HAT Domain	
				2PQ8	AcCoA	HAT Domain	
				2Y0M	AcCoA	HAT Domain	[332]
				3QAH		HAT Domain	[333]
				3TOA		HAT Domain	[334]
				3TOB		HAT Domain (mutate)	
			*Mus musculus*	1WGS		Tudor Domain	
		**ESA1**	*Saccharomyces cerevisiae*	1FY7	AcCoA	HAT Domain	[335]
				1MJ9	AcCoA	HAT Domain (mutate)	[336]
				1MJA	AcCoA	HAT Domain	
				1MJB	AcCoA	HAT Domain (mutate)	
				2RNZ		Chromodomain	[337]
				2RO0		Tudor Domain	
				3TO6	H4K16CoA	HAT Domain	[334]
				3TO7		HAT Domain	
				3TO9		HAT Domain (mutate)	
**Type B**		**HAT1**	*Homo sapiens*	2P0W	AcCoA	HAT	
			*Saccharomyces cerevisiae*	1BOB	AcCoA	HAT	[338]
		**Rtt109**	*Saccharomyces cerevisiae*	2RIM	AcCoA	HAT	[339]
				2ZFN	AcCoA	HAT	
				3CZ7	AcCoA	HAT	[340]
				3Q33	AcCoA	HAT	[341]
				3Q35	AcCoA	HAT	
				3Q66	AcCoA	Full length	[342]
				3Q68	AcCoA	Full length	
				3QM0	AcCoA	HAT	[343]

**Table 4.** Available Three-dimensional Structures of Mammals PKMTs

Name	Organism	PDB ID	Ligand	Domain	References
**DOT1L**	*Homo sapiens*	1NW3, 3QOW	SAM		[365,366]
		3QOX	SAH		[366]
		3SX0	brominated SAH analog		
		3SR4	TT8^a^		[367]
		3UWP	5-iodotubercidin		
**MLL1**	*Homo sapiens*	2W5Y	SAH	Methyltransferase	[368]
		2W5Z	Histone peptide, SAH	Methyltransferase	[368]
		2KU7		PHD3-Cyp33 RRM chimeric protein (NMR)	[369]
		3LPY		PHD3-bromo cassette	
		3LQH		Third PHD finger and bromo	
		3LQI	H3(1-9)K4me2 peptide	PHD3-bromo	
		2KYU	PHD3 finger	PHD3 finger	[370]
**EHMT1**	*Homo sapiens*	2IGQ	SAH	C-terminal	[371]
		2RFI	SAH	Catalytic	
		3B7B		Ankyrin repeat domains	[372]
		3B95	Histone H3 N-terminal Peptide	Ankyrin repeat	
		3FPD	BIX-01294^a^, SAH	SET	[373]
		3HNA	Mono-Methylated H3K9 Peptide SAH	Catalytic	[371]
		3MO0	E11^a^, SAH	SET	[364]
		3MO2	E67^a^, SAH	SET	
		3MO5	E72^a^, SAH	SET	
		3SW9	Dnmt3^a^ peptide, Sinefungin	C-terminal	[374]
		3SWC	Dnmt3^a^ peptide, SAH	C-terminal	[374]
**EHMT2**	*Homo sapiens*	2O8J	SAH	SET	[371]
		3K5K	DXQ^a^, SAH,	SET	[375]
		3RJW	CIQ^a^, SAH	SET	[376]
		3DM1			
**SUV39H1**	*Homo sapiens*	3MTS		Chromo	
**SUV39H2**	*Homo sapiens*	2R3A	SAM	Methyltransferase	[371]
**NSD1**	*Homo sapiens*	3OOI	SAM	SET	[377]
**WHSC1L1**	*Homo sapiens*	2DAQ		PWWP (NMR)	
**SETD1A**	*Homo sapiens*	3S8S		RRM	
**SETD3**	*Homo sapiens*	3SMT	SAM		
**SETD6**	*Homo sapiens*	3QXY	SAM	N-lysine methyltransferase	[378]
		3RC0	SAM	N-lysine methyltransferase	
**SETD7**	*Homo sapiens*	1H3I		N-terminal, SET	[379]
		1MT6	SAH	N-terminal, SET	[380]
		1MUF			
		1N6A	SAM	SET	[381]
		1N6C			
		1O9S	SAH, N-methyl-lysine	N-terminal, SET	[382]
		1XQH	p53 peptide, SAH, N-methyl-lysine	N-terminal, SET	[383]
		2F69	TAF10 peptide, SAH, N-methyl-lysine	N-terminal, SET	[384]
		3CBM	Estrogen receptor peptide, SAH	SET	[385]
		3CBO	Estrogen receptor peptide, SAH		
		3CBP	Estrogen receptor peptide, SAH, Sinefungin		
		3M53, 3M54, 3M55, 3M56, 3M57, 3M58, 3M59, 3M5A	TAF peptide, SAH	SET with various mutations	
		3OS5	Dnmt1 peptide, SAH, N-methyl-lysine	SET	[386]
		4E47	SAM, 0N6^a^	SET	
**SETD8**	*Homo sapiens*	1ZKK	Histone 4 peptide, SAH	SET	[387]
		2BQZ	Histone 4 peptide, SAH, N-methyl-lysine	SET	[388]
		3F9W	Histone 4 peptide, SAH	SET (Y334F)	[389]
		3F9X	Histone 4 peptide, SAH, N-dimethyl-lysine	SET (Y334F)	
		3F9Y	Histone 4 peptide, SAH, N-methyl-lysine	SET (Y334F)	
		3F9Z	Histone 4 peptide, SAH	SET (Y334F)	
**SETD2**	*Homo sapiens*	2A7O		HSET2/HYBP SRI (NMR)	[390]
		3H6L	SAM	SET	
**SETDB1**	*Homo sapiens*	3DLM		Tudor	
**SETMAR**	*Homo sapiens*	3BO5	SAH	N-methyltransferase	
		3F2K	LTFA peptide, selenomethionine	Transposase	
		3K9J		Transposase	[391]
		3K9K		Transposase	
**ASH1L**	*Homo sapiens*	3MQM		Bromo	[294]
		3OPE	SAM	SET	
**SUV420H1**	*Homo sapiens*	3S8P	SAM, selenomethionine	SET	
**SUV420H2**	*Homo sapiens*	3RQ4	SAM	SET	
**SMYD1**	*Mus musculus*	3N71	Sinefungin	SET and MYND	[392]
**SMYD2**	*Mus musculus*	3QWV	SAH, Sinefungin	SET and MYND	[393]
		3QWW			
	*Homo sapiens*	3RIB	SAH	SET and MYND	[394]
		3S7B	NH5^a^, SAM	SET and MYND	[395]
		3S7D	Monomethylated p53 peptide, SAH		
		3S7F	p53 peptide, SAM		
		3S7J	SAM		
		3TG4	SAM	SET and MYND	[396]
		3TG5	p53 peptide, SAH		
**SMYD3**	*Homo sapiens*	3MEK	Selenomethionine, SAM	SET and MYND	
		3OXF	SAH	SET and MYND	[397]
		3OXG			
		3OXL			
		3PDN	Sinefungin	SET and MYND	[398]
		3QWP	SAM	SET and MYND	
		3RU0	Sinefungin	SET and MYND	[399]
**PRDM1**	*Homo sapiens*	3DAL		SET	
**PRDM2**	*Homo sapiens*	2JV0		SET (NMR)	[400]
		2QPW		SET	[371]
**PRDM4**	*Homo sapiens*	2L9Z		Residues 366-402	[401]
		3DB5	Selenomethionine	SET	
**PRDM10**	*Homo sapiens*	3IHX		SET	
**PRDM11**	*Homo sapiens*	3RAY		SET	
**PRDM12**	*Homo sapiens*	3EP0		SET	

a ligand PDB ID

**Table 5.** Available Three-dimensional Structures of Mammals PRMTs

Name	Organism	PDB ID	Ligand	Domain	References
**CARM1**	*Rattus norvegicus*	2OQB		N-terminal	[402]
		3B3F	SAH	Catalytic	
		3B3G		Catalytic	
		3B3J		N-terminal, Catalytic and C-terminal	
	*Mus musculus*	2V74	SAH	Catalytic	[404]
		2V7E			
	*Homo sapiens*	2Y1W	Sinefungin, 849^a^	Catalytic	[403]
		2Y1X	SAH, 845^a^		
**PRMT1**	*Rattus norvegicus*	1OR8	Substrate peptide, SAH	Full length	[405]
		1ORH	Substrate peptide, SAH	Full length (E153Q)	
		1ORI	SAH	Full length	
		3Q7E	SAH	Full length	
**PRMT2**	*Homo sapiens*	1X2P		SH3	
**PRMT3**	*Mus musculus*	1WIR		C2H2 zinc finger	
	*Rattus norvegicus*	1F3L	SAH	C2H2 zinc finger, SAM binding and catalytic	[406]
	*Homo sapiens*	2FYT	SAH	SAM binding and catalytic	
		3SMQ	TDU^a^	SAM binding and catalytic	
**ECE2**	*Homo sapiens*	2PXX	SAH	Methyltransferase-like region (R100C)	[407]
**METTL11A**	*Homo sapiens*	2EX4	SAH	Full length	

a ligand PDB ID

**Table 6.** Available Three-dimensional Structures of Mammals DNMTs

Name	Organism	PDB ID	Ligand	Domain	References
**DNMT1**	*Mus musculus*	3AV4		DNMT1	
		3AV5	SAH	DNMT1	
		3AV6	SAM	DNMT1	
		3PT6	DNA, SAH	DNMT1	[414]
		3PT9	SAH	DNMT1 and DNA complex	
	*Homo sapiens*	3EPZ	RFTS domain, Beta-d-glucose	DNMT1	[415]
		3PTA	DNA, SAH	RFTS	[414]
		3SWR	Sinefungin, MES^a^,	DNMT1 and DNA complex	
		3OS5	SETD7, SAH	DNMT1	[386]
**DNMT2**	*Homo sapiens*	1G55	SAH	Complex with SETD7	[412]
**DNMT3A**	*Homo sapiens*	2QRV		DMNT2 (deleted in 191-237)	[416]
		3A1A		DNMT3a-DNMT3L C-terminal complex	[417]
		3A1B		ADD and histone H3 complex	
		3LLR		ADD and histone H3 complex	[418]
**DNMT3B**	*Mus musculus*	1KHC		PWWP	[419]
	*Homo sapiens*	3FLG		PWWP	
		3QKJ		PWWP	[418]
**DNMT3L**	*Homo sapiens*	2PV0		PWWP	[420]
		2PVC	Histone H3 peptide	DNMT3L	
		2QRV	SAH	DNMT3L - DNMT3a C-terminal complex	[416]

a ligand PDB ID

**Table 7.** Available Three-dimensional Structures of Demethylases

Name	Organism	PDB ID	Ligand	Domain	References
**LSD1**	*Homo sapiens*	2COM	-	SWIRM	[432]
		2DW4	FAD		[433]
		2EJR	F2N^a^		
		2Z3Y	F2N^a^	Full length	
		2Z5U	FA9^a^		
		2H94	FAD		[434]
		2HKO	FAD		[435]
		2IW5	CoREST 1 peptide, FAD	SWIRM, amine oxidase and linker	[436]
		2L3D		SWIRM domain	
		2UXN	CoREST 1 peptide, Histone H3 peptide, FDA^a^	SWIRM domain, amine oxidase domain and linker	[437]
		2UXX	CoREST 1 peptide, Histone H3 peptide, FA9^a^		
		2V1D	CoREST 1 peptide, Histone H3 peptide, FAD	SWIRM domain, amine oxidase domain and linker	[438]
		2X0L	CoREST 1 peptide, Histone H3 peptide, FAD	Full length	[439]
		2XAF	CoREST 1 peptide, FAD, TCF^a^	Full length	[440]
		2XAG			
		2XAH	CoREST 1 peptide, FAD, 3PL^a^		
		2XAJ	CoREST 1 peptide, FAD, TCA^a^		
		2XAQ	CoREST 1 peptide, FAD, M84^a^		
		2XAS	CoREST 1 peptide, FAD, M80^a^		
		2Y48	CoREST 1 peptide, Zinc finger protein SNAI1, FAD	Full length	[441]
		3ABT	amine oxidase domain 2, 2PF^a^	Full length	[442]
		3ABU	amine oxidase domain 2, 12F^a^		
**JMJD5**	*Homo sapiens*	3UYJ	AKG^c^	JmjC	
		4AAP	OGA^b^	JmjC	
**JMJD6**	*Homo sapiens*	3K2O	-	Full length	[443]
		3LD8	antibody Fab fragment	Full length	
		3LD^b^	antibody Fab fragment, AKG^c^	Full length	
**FBXL11**	*Homo sapiens*	2YU1	AKG^c^	JmjC	
		2YU2	-		
**JHDM1D**	*Homo sapiens*	3KV5	OGA^b^	JmjC	[444]
		3KV6	AKG^c^		
		3KV9	-		
		3KV^a^	AKG^c^		
		3KV^b^	OGA^b^		
		3U78	AKG, E67^a^	JmjC	[445]
**PHF8**	*Caenorhabditis elegans*	3N9L	Histone H3 peptide, OGA^b^	PHD and JmjC	[446]
		3N9M	-	PHD	
		3N9N	Histone H3 peptide, OGA^b^	PHD and JmjC	
		3N9O	Histone H3 peptides, OGA^b^	PHD and JmjC	
		3N9P	Histone H3 peptide, OGA^b^	PHD and JmjC	
		3N9Q	Histone H3 peptides, OGA^b^	PHD and JmjC	
		3PUQ	AKGc	PHD	[447]
		3PUR	2HG^a^	PHD	
	*Mus musculus*	1WEP	-	PHD	
	*Homo sapiens*	2WWU	BGC^a^	PHD and JmjC	[448]
		3K3N	-	PHD	[449]
		3K3O	AKGc	PHD	
		3KV4	OGA^b^	PHD	[444]
**PHF2**	*Homo sapiens*	3KQI	Histone H3 peptide	PHD finger	[450]
		3PTR	-	JmjC	[451]
		3PU3 3PU8 3PUA 3PUS	OGA^b^	JmjC	
**JMJD3**	*Homo sapiens*	2XUE	AKG^c^	JmjC	
		2XXZ	8XQ^a^	JmjC	
**UTX**	*Homo sapiens*	3AVS	OGA^b^	JmjC	[452]
		3AVR	OGA^b^		
**JMJD2A**	*Homo sapiens*	2GF7	-	tudor	[453]
		2GF^a^	-		
		2GP3	-	JmjC	[454]
		2GP5	AKGc		
		2OQ6	OGA^b^	JmjC	[455]
		2OQ7	OGA^b^		
		2OS2	Histone H3 peptide, OGA^b^		
		2OT7	Histone H3 peptide monomethyl, OGA^b^		
		2OX0	synthetic peptide, OGA^b^		
		2P5^b^	Histone H3 peptide, OGA^b^	JmjC	[456]
		2PXJ	monomethylated Histone H3 peptide, OGA^b^		
		2Q8^c^	Histone H3 peptide, AKG^c^	JmjC	[457]
		2Q8D	Histone H3 peptide, SIN^a^		
		2Q8E	Histone H3 peptide, AKG^c^		
		2QQR	-	tudor	[458]
		2QQS	Histone H4 peptide		
		2VD7	PD2^a^	JmjC	
		2WWJ	Y28^a^	JmjC	[459]
		2YBK	2HG^a^	JmjC	[460]
		2YBP	Histone H3 peptide,2HG^a^		
		2YBS	Histone H3 peptide, S2G^a^		
		3NJY	8XQ^a^	JmjC	[461]
		3PDQ	KC6^a^	JmjC	[462]
		3U4S	T11C Peptide, 08P^a^	JmjC	[463]
**JMJD2C**	*Homo sapiens*	2XDP	-	tudor	
		2XML	OGA^b^	JmjC	
**JMJD2D**	*Homo sapiens*	3DXT	-	JmjC	
		3DXU	OGA^b^	JmjC	
**JARID1B**	*Mus musculus*	2EQY	-	ARID	
**JARID1C**	*Homo sapiens*	2JRZ	-	ARID	[464]
**JARID1D**	*Homo sapiens*	2E6R	-	PDH	
		2YQE	-	ARID	
**JARID1A**	*Homo sapiens*	2JXJ	-	ARID	[465]
		2KGG		C-terminal PHD finger	[466]
		2KGI	Histone H3 peptide		
		3GL6	Histone H3 peptide	C-terminal PHD finger	
**JARID2**	*Mus musculus*	2RQ5	-	ARID	[467]
**MINA**	*Homo sapiens*	2XDV	OGA^b^	JmjC	
**NO66**	*Homo sapiens*	4DIQ	PD2^a^	JmjC	

a PDB ligand ID

b N-Oxalylglycine

c α-Ketoglutaric acid

## ABBREVIATIONS

α-KG: = α-Ketoglutarate
AACR: = American Association for Cancer Research
AcCoA: = Acetyl coenzyme A
AML: = Acute myeloid leukemia
APAH: = Acetylpolyamine aminohydrolase
APL: = Acute promyelocytic leukemia
ARTC: = ADP-ribosyltransferase clostridial toxin-like
ARTD: = ADP-ribosyltransferase diphtheria toxin-like
ATP: = Adenosine triphosphate
BCP: = Bromodomain containing proteins
BD: = Bromodomains
CADD: = Computer-aided drug design
CARM1: = Coactivator-associated arginine methyltransferase
CBP: = (cAMP-responsive element binding protein)-binding protein
CLL: = Chronic lymphocytic leukemia
CML: = Chronic myeloid leukaemia
CoREST: = Corepressor RE1 silencing transcription
CpG: = Cytosine-phosphate-guanine
CREB: = cAMP-responsive element binding protein
CRC: = Colorectal cancer
DOT1: = Disruptor of telomeric silencing 1
DNA: = Desoxyribonucleic acid
DNMT: = DNA methyltransferase
DUB: = Deubiquitinating enzymes
EGFR: = Epidermal growth factor receptor
ER: = Estrogen receptor
EZH2: = Enhancer of zeste homolog 2
FAD: = Flavin adenine dinucleotide
Gcn5: = General Control non-derepressible 5
GNATs: = (General Control non-derepressible 5)-related N-acetyltransferases
HAT: = Histone acetyltransferases
HBD: = Histone binding domains
HBV: = Hepatitis B virus
HCV: = Hepatitis C virus
HCC: = Hepatocellular carcinoma
HDAC: = Histone deacetylases
HDACi: = Histone deacetylase inhibitors
HDAH: = Histone deacetylase-like amidohydrolase
HDLP: = Histone deacetylase-like protein
HER2: = Human epidermal growth factor 2
HMT: = Histone methyltransferases
HTS: = High-throughtput screening
miRNA: = Micro RNA
mRNA: = Messenger RNA
MLL: = Mixed-lineage leukemia
NAD+/NADH: = Nicotinamide adenine dinucleotide
NADP+/NADPH: = Nicotinamide adenine dinucleotide phosphate
NHL: = Non-Hodgkin’s Lymphoma
NSCLCs: = Non-small-cell lung carcinoma
NUT: = Nuclear protein in testis
OGA: = O-GlcNAcase
O-GlcNAc: = O-linked N-acetylglucosamine
OGT: = O-GlcNAc transferase
PAD: = protein-arginine deiminase
PARP: = Poly ADP-ribose polymerase
PKMT: = Protein lysine methyltransferase
piRNA: = Piwi-interacting RNA
Piwi: = P- element-induced wimpy testes
PcG: = Polycomb
PMTs: = Protein methyltransferases
PR: = Progesterone receptor
PRC2: = Polycomb repressive complex
PRMT: = Protein arginine mehyltransferase
PTM: = Post-translational modifications
RCC: = Renal cell carcinomas
RCS: = Reactive carbonyl species
RISC: = RNA-induced silencing complex
RNA: = Ribonucleic acid
SAH: = S-adenosyl homocysteine
SAHA: = Suberoylanilide hydroxamic acid (Vorinostat)
SAM: = S-adenosyl methionine
SET: = Su(Var)3-9, Enhancer of zeste, Trithirax
siRNA: = Small interfering RNA
Ub: = Ubiquitin
UBA: = Ubiquitin-activating (UBA) enzyme
UDP: = Uridine 5'-diphosphate
UPS: = Ubiquitin-proteasome system

## References

[1]  Martin C, Zhang Y (2007). Mechanisms of epigenetic inheritance. Curr opinion cell Biol.

[2]  Herceg Z, Ushijima T (2010). Introduction: epigenetics and cancer.

[3] Baylin SB (2008). Epigenetics and Cancer. The Molecular Basis of Cancer.

[4]  Jones P a, Baylin SB (2007). The epigenomics Cancer. Cell.

[5]  Kouzarides T (2007). Chromatin modifications and their function. Cell.

[6]  Kelly TK, De Carvalho DD, Jones P a (2010). Epigenetic modifications as therapeutic targets. Nature Biotechnol.

[7]  Gardner KE, Allis CD, Strahl BD (2011). Operating on chromatin, a colorful language where context matters. J Mol Biolo.

[8]  Sidoli S, Cheng L, Jensen ON (2012). Proteomics in chromatin biology and epigenetics: Elucidation of post-translational modifications of histone proteins by mass spectrometry. J Proteomics.

[9]  Cosgrove MS, Boeke JD, Wolberger C (2004). Regulated nucleosome mobility and the histone code. Nature structural Mol Biol.

[10]  Cosgrove MS, Wolberger C (2005). How does the histone code work?. Biochemistry and cell biology = Biochimie et biologie cellulaire.

[11]  Cruickshank MN, Besant P, Ulgiati D (2010). The impact of histone posttranslational modifications on developmental gene regulation. Amino acids.

[12]  Bannister AJ, Kouzarides T (2011). Regulation of chromatin by histone modifications. Cell Res.

[13]  Imhof A (2006). Epigenetic regulators and histone modification. Briefings Functional Genomics Proteomics.

[14]  Mellor J, Dudek P, Clynes D (2008). A glimpse into the epigenetic landscape of gene regulation. Curr opinion in genetics &development.

[15]  Weake VM, Workman JL (2008). Histone ubiquitination: triggering gene activity. Molecular cell.

[16]  Loyola A, Almouzni G (2007). Marking histone H3 variants: how, when and why?. Trends in Biochem Sci.

[17]  De Koning L, Corpet A, Haber JE, Almouzni G (2007). Histone chaperones: an escort network regulating histone traffic. Nature Structural Mol Biol.

[18]  Suganuma T, Workman JL (2008). Crosstalk among Histone Modifications. Cell.

[19]  Sippl W, Jung M (2009). Epigenetic targets in drug discovery.

[20]  Chi P, Allis CD, Wang GG (2010). Covalent histone modifications--miswritten, misinterpreted and mis-erased in human cancers. Nat Rev Cancer.

[21]  Santos-Rosa H, Caldas C (2005). Chromatin modifier enzymes the histone code and cancer. Eur J cancer (Oxford England: 1990).

[22]  Golbabapour S, Abdulla MA, Hajrezaei M (2011). A concise review on epigenetic regulation: insight into molecular mechanisms.

[23]  Rius M, Lyko F (2011). Epigenetic cancer therapy: rationales, targets and drugs. Oncogene.

[24]  Copeland R a, Olhava EJ, Scott MP (2010). Targeting epigenetic enzymes for drug discovery. Cur opinion Chem Biol.

[25]  Dhanak D (2012). Cracking the Code: The Promise of Epigenetics. ACS Medicinal Chemistry Letters.

[26]  Cairns R a, Harris IS, Mak TW (2011). Regulation of cancer cell metabolism. Nat Rev Cancer.

[27]  Gerhäuser C (2012). Cancer cell metabolism , epigenetics and the potential influence of dietary components - A perspective. Biomedical Res.

[28]  Semenza GL (2011). A return to cancer metabolism. J molecular medicine (Berlin, Germany).

[29]  Galluzzi L, Senovilla L, Zitvogel L, Kroemer G (2012). The secret ally: immunostimulation by anticancer drugs. Nat Rev Drug Discov.

[30]  Cavallo F, De Giovanni C, Nanni P, Forni G, Lollini P-L (2011). 2011: the immune hallmarks of cancer. Cell.

[31]  Ellis L, Atadja PW, Johnstone RW (2009). Epigenetics in cancer: targeting chromatin modifications. Molecular cancer therapeutics.

[32]  Altucci L, Minucci S (2009). Epigenetic therapies in haematological malignancies: searching for true targets. Eur J cancer (Oxford, England: 1990).

[33]  Herranz M, Esteller M (2006). New therapeutic targets in cancer: the epigenetic connection. Clinical &translational oncology??: official publication of the Federation of Spanish Oncology Societies and of the National Cancer Institute of Mexico.

[34]  Graham JS, Kaye SB, Brown R (2009). The promises and pitfalls of epigenetic therapies in solid tumours. Eur J cancer (Oxford, England:1990).

[35]  Rodríguez-Paredes M, Esteller M (2011). Cancer epigenetics reaches mainstream oncology. Nature medicine.

[36]  Kulis M, Esteller M (2010). DNA methylation and cancer. Advances in genetics.

[37]  Meeran SM, Ahmed A, Tollefsbol TO (2010). Epigenetic targets of bioactive dietary components for cancer prevention and therapy. Clinical epigenetics.

[38]  Ljungman M (2009). Targeting the DNA damage response in cancer. Chemical reviews.

[39]  Claes B, Buysschaert I, Lambrechts D (2010). Pharmaco-epigenomics: discovering therapeutic approaches and biomarkers for cancer therapy. Heredity.

[40]  Pollock RM, Richon VM (2009). Epigenetic approaches to cancer therapy. Drug Discov Today: Therapeutic Strategies.

[41]  Spannhoff A, Sippl W, Jung M (2009). Cancer treatment of the future: inhibitors of histone methyltransferases. The international J biochemistry &cell Biol.

[42]  Sala A, Corona DFV (2008). Epigenetics: More than genetics. Fly.

[43]  Best JD, Carey N (2010). Epigenetic opportunities and challenges in cancer. Drug Discov Today.

[44]  Lohrum M, Stunnenberg HG, Logie C (2007). The new frontier in cancer research: deciphering cancer epigenetics. The international J biochemistry &cell Biol.

[45]  Inche AG, La Thangue NB (2006). Chromatin control and cancer-drug discovery: realizing the promise. Drug Discov Today.

[46]  Boumber Y, Issa J-PJ (2011). Epigenetics in cancer: what’s the future?. Oncology (Williston Park, N.Y.).

[47]  Veeck J, Esteller M (2010). Breast cancer epigenetics: from DNA methylation to microRNAs. J mammary gland biology and neoplasia.

[48]  Cebrian A, Pharoah PD, Ahmed S (2006). Genetic variants in epigenetic genes and breast cancer risk. Carcinogenesis.

[49]  Lustberg MB, Ramaswamy B (2010). Epigenetic Therapy in Breast Cancer. Curr Breast Cancer Reports.

[50]  Bombonati A, Sgroi DC (2011). The molecular pathology of breast cancer progression. J pathology.

[51]  Caporuscio F, Rastelli G, Imbriano C, Del Rio A (2011). Structure-based design of potent aromatase inhibitors by high-throughput docking. J medicinal Chem.

[52]  Dutta U, Pant K (2008). Aromatase inhibitors: past, present and future in breast cancer therapy. Medical oncology (Northwood, London, England).

[53]  Geisler J (2011). Differences between the non-steroidal aromatase inhibitors anastrozole and letrozole--of clinical importance?. British J cancer.

[54]  Macedo LF, Sabnis G, Brodie A (2009). Aromatase inhibitors and breast cancer. Annals of the New York Academy of Sciences.

[55]  Kristensen LS, Nielsen HM, Hansen LL (2009). Epigenetics and cancer treatment. Eur J pharmacology.

[56]  Jovanovic J, Rønneberg JA, Tost J, Kristensen V (2010). The epigenetics of breast cancer. Molecular Oncol.

[57]  Trimarchi MP, Mouangsavanh M, Huang TH-M (2011). Cancer epigenetics: a perspective on the role of DNA methylation in acquired endocrine resistance. Chinese J cancer.

[58]  Billam M, Witt A (2009). The silent estrogen receptor. Cancer Biology.

[59]  Bièche I, Lidereau R (2011). Genome-based and transcriptome-based molecular classification of breast cancer. Curr opinion Oncol.

[60]  Rizzolo P, Silvestri V, Falchetti M (2011). Inherited and acquired alterations in development of breast cancer. The Application of Clinical.

[61]  De Santa F, Iosue I, Del Rio A, Fazi F (2013). microRNA biogenesis pathway as a therapeutic target for human disease and cancer. Curr Pharm Des.

[62]  Velkova A, Monteiro AN a (2011). Epigenetic tumor suppression by BRCA1. Nature medicine.

[63]  Cai F-F, Kohler C, Zhang B, Wang M-H, Chen W-J, Zhong X-Y (2011). Epigenetic therapy for breast cancer. International J molecular sciences.

[64]  Hegi ME, Sciuscio D, Murat A, Levivier M, Stupp R (2009). Epigenetic deregulation of DNA repair and its potential for therapy. Clinical cancer research: an official J Am Ass Cancer Res.

[65]  Jones P (2012). Development of second generation epigenetic agents. MedChemComm.

[66]  Khan SI, Aumsuwan P, Khan I a, Walker L a, Dasmahapatra AK (2012). Epigenetic events associated with breast cancer and their prevention by dietary components targeting the epigenome. Chemical research in toxicology.

[67]  Kurebayashi J (2005). Resistance to endocrine therapy in breast cancer. Cancer chemotherapy and pharmacology.

[68] Nabholtz JM (2008). Aromatase inhibitors in the management of early breast cancer. Eur J surgical oncology: J Eur Society of Surgical Oncology and the British Association of Surgical Oncol.

[69]  Cuzick J (2008). Aromatase inhibitors in early breast-cancer treatment: The story so far. Breast (Edinburgh, Scotland).

[70]  Yoo KH, Hennighausen L (2012). EZH2 methyltransferase and H3K27 methylation in breast cancer. International J biological sciences.

[71]  Stefansson OA, Esteller M (2011). EZH2-mediated epigenetic repression of DNA repair in promoting breast tumor initiating cells. Breast cancer research: BCR.

[72]  Copeland R a (2011). Protein methyltransferase inhibitors as personalized cancer therapeutics. Drug Discov Today: Therapeutic Strategies.

[73]  Copeland R a, Solomon ME, Richon VM (2009). Protein methyltransferases as a target class for drug discovery. Nat Rev Drug Discov.

[74]  Géranton SM (2012). Targeting epigenetic mechanisms for pain relief. Curr opinion in pharmacology.

[75]  Su Y, Shankar K, Rahal O, Simmen RCM (2011). Bidirectional signaling of mammary epithelium and stroma: implications for breast cancer--preventive actions of dietary factors. J Nutritional BioChem.

[76]  Ramaswamy B, Sparano J a (2010). Targeting Epigenetic Modifications for the Treatment and Prevention of Breast Cancer. Curr Breast Cancer Reports.

[77]  Thornburg KL, Shannon J, Thuillier P, Turker MS (2010). In utero life and epigenetic predisposition for disease.

[78]  Feinberg AP, Vogelstein B (1983). Hypomethylation distinguishes genes of some human cancers from their normal counterparts. Nature.

[79]  Kim YS, Deng G (2007). Epigenetic changes (aberrant DNA methylation) in colorectal neoplasia. Gut and liver.

[80]  Kondo Y, Issa J-PJ (2004). Epigenetic changes in colorectal cancer. Cancer metastasis reviews.

[81]  Grady WM (2004). Genomic instability and colon cancer. Cancer metastasis reviews.

[82]  Grady WM, Markowitz SD (2002). Genetic and epigenetic alterations in colon cancer. Annual review of genomics and human genetics.

[83]  Grady WM (2005). Epigenetic events in the colorectum and in colon cancer. Biochemical Society transactions.

[84]  Carmona FJ, Esteller M (2010). Epigenomics of human colon cancer. Mutation Res.

[85]  Kim MS, Lee J, Sidransky D (2010). DNA methylation markers in colorectal cancer. Cancer metastasis reviews.

[86]  Samowitz WS (2008). Genetic and epigenetic changes in colon cancer. Experimental and molecular pathology.

[87]  Grady WM, Carethers JM (2008). Genomic and epigenetic instability in colorectal cancer pathogenesis. Gastroenterology.

[88]  Garagnani P, Pirazzini C, Franceschi C (2013). Colorectal Cancer Microenvirnoment: between Nutrition, Gut Microbiota, Inflammation and Epigenetics. Curr Pharm Des.

[89]  Slattery ML, Wolff RK, Curtin K (2009). Colon tumor mutations and epigenetic changes associated with genetic polymorphism: insight into disease pathways. Mutation Res.

[90]  van Engeland M, Herman JG (2010). Viewing the epigenetics of colorectal cancer through the window of folic acid effects. Cancer prevention research (Philadelphia, Pa.).

[91]  Nyström M (2009). Diet and epigenetics in colon cancer. World J Gastroenterology.

[92]  Giardina C, Madigan JP, Tierney CAG, M Brenner B, Rosenberg DW (2012). Vitamin D resistance and colon cancer prevention. Carcinogenesis.

[93]  Cho WCS (2011). Epigenetic alteration of microRNAs in feces of colorectal cancer and its clinical significance. Expert review of molecular diagnostics.

[94]  Pucci S, Mazzarelli P (2011). MicroRNA Dysregulation in Colon Cancer Microenvironment Interactions: The Importance of Small Things in Metastases. Cancer microenvironment: official J Int Cancer Microenvironment Society.

[95]  Kalimutho M, Di Cecilia S, Del Vecchio Blanco G (2011). Epigenetically silenced miR-34b/c as a novel faecal-based screening marker for colorectal cancer. British J cancer.

[96]  Kunej T, Godnic I, Ferdin J, Horvat S, Dovc P, Calin GA (2011). Epigenetic regulation of microRNAs in cancer: an integrated review of literature. Mutation Res.

[97]  Duthie SJ (2011). Epigenetic modifications and human pathologies: cancer and CVD. The Proceedings of the Nutrition Society.

[98]  Mariadason JM (2008). HDACs and HDAC inhibitors in colon cancer. Epigenetics: official J DNA Methylation Society.

[99]  Wilson AJ, Chueh AC, Tögel L (2010). Apoptotic sensitivity of colon cancer cells to histone deacetylase inhibitors is mediated by an Sp1/Sp3-activated transcriptional program involving immediateearly gene induction. Cancer Res.

[100]  Nosho K, Shima K, Irahara N (2009). SIRT1 histone deacetylase expression is associated with microsatellite instability and CpG island methylator phenotype in colorectal cancer. Modern pathology􀀁:
an official J United States and Canadian Academy of Pathology,
Inc.

[101]  Chou C-W, Wu M-S, Huang W-C, Chen C-C (2011). HDAC inhibition decreases the expression of EGFR in colorectal cancer cells. PloS one.

[102]  Bishton M, Kenealy M, Johnstone R, Rasheed W, Prince HM (2007). Epigenetic targets in hematological malignancies: combination therapies with HDACis and demethylating agents. Expert review of anticancer therapy.

[103]  Petrie K, Zelent A, Waxman S (2009). Differentiation therapy of acute myeloid leukemia: past, present and future. Curr opinion in hematology.

[104]  Florean C, Schnekenburger M, Grandjenette C, Dicato M, Diederich M (2011). Epigenomics of leukemia: from mechanisms to therapeutic applications. Epigenomics.

[105]  Masetti R, Serravalle S, Biagi C, Pession A (2011). The role of HDACs inhibitors in childhood and adolescence acute leukemias. J biomedicine &biotechnology.

[106]  Downing JR (2008). Targeted therapy in leukemia. Modern pathology􀀁: an
official J United States and Canadian Academy of Pathology, Inc.

[107]  Abujamra AL, Dos Santos MP, Roesler R, Schwartsmann G, Brunetto AL (2010). Histone deacetylase inhibitors: a new perspective for the treatment of leukemia. Leukemia Res.

[108]  Bug G, Ottmann OG (2010). The DAC system and associations with acute leukemias and myelodysplastic syndromes. Investigational new drugs.

[109]  Daigle SR, Olhava EJ, Therkelsen C a (2011). Selective killing of mixed lineage leukemia cells by a potent small-molecule DOT1L inhibitor. Cancer cell.

[110]  Bernt KM, Zhu N, Sinha AU (2011). MLL-rearranged leukemia is dependent on aberrant H3K79 methylation by DOT1L. Cancer cell.

[111]  Zeisig BB, Cheung N, Yeung J, So CWE (2008). Reconstructing the disease model and epigenetic networks for MLL-AF4 leukemia. Cancer cell.

[112]  Oka T, Sato H, Ouchida M, Utsunomiya A, Yoshino T (2011). Cumulative Epigenetic Abnormalities in Host Genes with Viral and Microbial Infection during Initiation and Progression of Malignant Lymphoma/Leukemia. Cancers.

[113]  Travers J, Blagg J, Workman P (2011). Epigenetics: Targeting leukemia on the DOT. Nature chemical Biol.

[114]  Geyer CR (2010). Strategies to re-express epigenetically silenced p15(INK4b) and p21(WAF1) genes in acute myeloid leukemia. Epigenetics: official J DNA Methylation Society.

[115]  Jain N, Rossi A, Garcia-Manero G (2009). Epigenetic therapy of leukemia: An update. The international J biochemistry &cell Biol.

[116]  Melnick AM (2010). Epigenetics in AML. Best practice &research. Clinical haematology.

[117]  Schoofs T, Müller-Tidow C (2011). DNA methylation as a pathogenic event and as a therapeutic target in AML. Cancer Treatment Rev.

[118]  Voso MT, D’Alò F, Greco M (2010). Epigenetic changes in therapyrelated MDS/AML. Chemico-biological interactions.

[119]  Chen J, Odenike O, Rowley JD (2010). Leukaemogenesis: more than mutant genes. Nat Rev Cancer.

[120]  Fathi AT, Abdel-Wahab O (2012). Mutations in epigenetic modifiers in myeloid malignancies and the prospect of novel epigenetic-targeted therapy. Adv Hematol.

[121]  Issa J-PJ, Kantarjian HM (2009). Targeting DNA methylation. Clinical cancer research: an official J Am Ass Cancer Res.

[122]  Poetsch AR, Plass C (2011). Transcriptional regulation by DNA methylation. Cancer Treatment Rev.

[123]  Wu M, Shu H-B (2011). MLL1/WDR5 complex in leukemogenesis and epigenetic regulation. Chinese J Cancer.

[124]  Klauke K, de Haan G (2011). Polycomb group proteins in hematopoietic stem cell aging and malignancies. Int J hematol.

[125]  Bernt KM, Armstrong SA (2011). A role for DOT1L in MLL-rearranged leukemias. Epigenomics.

[126]  Bullinger L, Armstrong S a (2010). HELP for AML: methylation profiling opens new avenues. Cancer Cell.

[127]  Schotte D, Pieters R, Den Boer ML (2012). MicroRNAs in acute leukemia: from biological players to clinical contributors. MicroRNAs in acute leukemia:
from biological players to clinical contributors. Leukemia􀀁: official
J Leukemia Society of America, Leukemia Research Fund,
U.K.

[128]  Ansari KI, Mandal SS (2010). Mixed lineage leukemia: roles in gene expression, hormone signaling and mRNA processing. FEBS J.

[129]  Tsiftsoglou AS, Bonovolias ID, Tsiftsoglou S a (2009). Multilevel targeting of hematopoietic stem cell self-renewal, differentiation and apoptosis for leukemia therapy. Pharmacol Therapeut.

[130]  Garcia-Manero G (2008). Demethylating agents in myeloid malignancies. Curr opinion Oncol.

[131]  Yu MK (2006). Epigenetics and chronic lymphocytic leukemia. Ame J Hematol.

[132]  Holloway a F, Oakford PC (2007). Targeting epigenetic modifiers in cancer. Curr medicinal Chem.

[133]  Heider U, von Metzler I, Kaiser M (2008). Synergistic interaction of the histone deacetylase inhibitor SAHA with the proteasome inhibitor bortezomib in mantle cell lymphoma. Eur J Haematol.

[134]  Nawrocki ST, Carew JS, Maclean KH (2008). Myc regulates aggresome formation, the induction of Noxa, and apoptosis in response to the combination of bortezomib and SAHA. Blood.

[135] Cancer’s epicentre [Internet]. 2012; Available from: http://www.economist.com/node/21552168.

[136]  Mercurio C, Minucci S, Pelicci PG (2010). Histone deacetylases and epigenetic therapies of hematological malignancies. Pharmacological research: the official J Italian Pharmacol Soc.

[137]  Mahadevan D, Fisher RI (2011). Novel therapeutics for aggressive non-
Hodgkin’s lymphoma. J clinical oncology: official J Am Soc Clin Oncol.

[138]  Zain J, O’Connor O a (2010). Targeting histone deacetyalses in the treatment of B- and T-cell malignancies. Investigational New Drugs.

[139]  Cotto M, Cabanillas F, Tirado M, García MV, Pacheco E (2010). Epigenetic therapy of lymphoma using histone deacetylase inhibitors. Clin Translational Oncol.

[140]  Hayslip J, Montero A (2006). Tumor suppressor gene methylation in follicular lymphoma: a comprehensive review. Mol Cancer.

[141]  Cang S, Ma Y, Liu D (2009). New clinical developments in histone deacetylase inhibitors for epigenetic therapy of cancer. J Hematol Oncol.

[142]  Ellis L, Pan Y, Smyth GK (2008). Histone deacetylase inhibitor panobinostat induces clinical responses with associated alterations in gene expression profiles in cutaneous T-cell lymphoma. Clinical cancer research: an official J Am Ass Cancer Res.

[143]  Esteller M (2003). Profiling aberrant DNA methylation in hematologic neoplasms: a view from the tip of the iceberg. Clin Immunol.

[144]  Muegge K, Young H, Ruscetti F, Mikovits J (2003). Epigenetic control during lymphoid development and immune responses: aberrant regulation, viruses, and cancer. Ann New York Academy Sci.

[145]  Claus R, Lübbert M (2003). Epigenetic targets in hematopoietic malignancies. Oncogene.

[146]  Yoshimi A, Kurokawa M (2011). Key roles of histone methyltransferase and demethylase in leukemogenesis. J Cellular Biochem.

[147]  Yang IV, Schwartz D a (2011). Epigenetic control of gene expression in the lung. Ame J Respiratory Critical Care Med.

[148]  Lu F, Zhang H-T (2011). DNA methylation and nonsmall cell lung cancer. Anatomical record (Hoboken, N.J.2007).

[149]  Heller G, Zielinski CC, Zöchbauer-Müller S (2010). Lung cancer: from single-gene methylation to methylome profiling. Cancer Metastasis Rev.

[150]  Cho WCS (2010). MicroRNAs as therapeutic targets for lung cancer. Zhongguo fei ai za zhi = Chinese J lung cancer.

[151]  Sekido Y, Fong KM, Minna JD (2003). Molecular genetics of lung cancer. Ann Rev Med.

[152]  Herman JG (2004). Epigenetics in lung cancer: focus on progression and early lesions. Chest.

[153]  Esteller M (2007). Cancer epigenomics: DNA methylomes and histonemodification maps. Nat Rev Genetics.

[154]  Brambilla E, Gazdar a (2009). Pathogenesis of lung cancer signaling pathways: roadmap for therapies. The European respiratory journal: official J Eur Soc Clin Respiratory Physiol.

[155]  Chan LW, Wang FF, Cho WC (2012). Genomic Sequence Analysis of EGFR Regulation by MicroRNAs in Lung Cancer. Curr Topics Med Chem.

[156]  Scott BR, Belinsky SA, Leng S, Lin Y, Wilder JA, Damiani LA (2009). Radiation-stimulated epigenetic reprogramming of adaptiveresponse genes in the lung: an evolutionary gift for mounting adaptive protection against lung cancer. Dose-response: a publication of Int Hormesis Soc.

[157]  Kim GH, Ryan JJ, Marsboom G, Archer SL (2011). Epigenetic mechanisms of pulmonary hypertension. Pulmonary circulation.

[158]  Juergens R a, Wrangle J, Vendetti FP (2011). Combination Epigenetic Therapy Has Efficacy in Patients with Refractory Advanced Non-Small Cell Lung Cancer. Cancer Discov.

[159]  Gridelli C, Rossi A, Maione P (2008). The potential role of histone deacetylase inhibitors in the treatment of non-small-cell lung cancer. Critical Rev oncol/hematol.

[160]  Tiseo M, Franciosi V, Ardizzoni a (2006). Multi-target inhibitors in nonsmall cell lung cancer (NSCLC). Annals of oncology: official J Eur Society Medical Oncology / ESMO.

[161]  Matei DE, Nephew KP (2010). Epigenetic therapies for chemoresensitization of epithelial ovarian cancer. Gynecologic Oncol.

[162]  Asadollahi R, Hyde C a C, Zhong XY (2010). Epigenetics of ovarian cancer: from the lab to the clinic. Gynecologic Oncol.

[163]  Balch C, Fang F, Matei DE, Huang TH-M, Nephew KP (2009). Minireview: epigenetic changes in ovarian cancer. Endocrinology.

[164]  Berry NB, Bapat S a (2008). Ovarian cancer plasticity and epigenomics in the acquisition of a stem-like phenotype. J ovarian Res.

[165]  Barton C a, Hacker NF, Clark SJ, O’Brien PM (2008). DNA methylation changes in ovarian cancer: implications for early diagnosis, prognosis and treatment. Gynecologic Oncol.

[166]  Balch C, Huang TH-M, Brown R, Nephew KP (2004). The epigenetics of ovarian cancer drug resistance and resensitization. Ame J obstetrics and gynecology.

[167]  Ahluwalia a, Yan P, Hurteau J a (2001). DNA methylation and ovarian
cancer. I. Analysis of CpG island hypermethylation in human
ovarian cancer using differential methylation hybridization. Gynecologic Oncol.

[168]  Ahluwalia a, Hurteau J a, Bigsby RM, Nephew KP (2001). DNA methylation
in ovarian cancer. II. Expression of DNA methyltransferases in
ovarian cancer cell lines and normal ovarian epithelial cells. Gynecologic Oncol.

[169]  Maradeo ME, Cairns P (2011). Translational application of epigenetic alterations: ovarian cancer as a model. FEBS letters.

[170]  Balch C, Matei DE, Huang TH-M, Nephew KP (2010). Role of epigenomics in ovarian and endometrial cancers. Epigenomics.

[171]  Takai N, Narahara H (2010). Histone deacetylase inhibitor therapy in epithelial ovarian cancer. J Oncol.

[172]  Takai N, Narahara H (2007). Human endometrial and ovarian cancer cells: histone deacetylase inhibitors exhibit antiproliferative activity, potently induce cell cycle arrest, and stimulate apoptosis. Curr medicinal Chem.

[173]  Chin SP, Dickinson JL, Holloway AF (2011). Epigenetic regulation of prostate cancer. Clinical Epigenetics.

[174]  Detchokul S, Frauman AG (2011). Recent developments in prostate cancer biomarker research: therapeutic implications. British J clinical pharmacology.

[175]  Albany C, Alva AS, Aparicio AM (2011). Epigenetics in prostate cancer. Prostate cancer.

[176]  Perry AS, Watson RWG, Lawler M, Hollywood D (2010). The epigenome as a therapeutic target in prostate cancer. Nat Rev Urology.

[177]  Reynolds M a (2008). Molecular alterations in prostate cancer. Cancer letters.

[178]  Pethe VV, Bapat B (2008). Molecular Genetic Etiology of Prostate Cancer. The Open Genomics J.

[179]  Dobosy JR, Roberts JLW, Fu VX, Jarrard DF (2007). The expanding role of epigenetics in the development, diagnosis and treatment of prostate cancer and benign prostatic hyperplasia. J urology.

[180]  Nakayama M, Gonzalgo ML, Yegnasubramanian S, Lin X, De Marzo AM, Nelson WG (2004). GSTP1 CpG island hypermethylation as a molecular biomarker for prostate cancer. J Cellular Biochem.

[181]  Henrique R, Jerónimo C (2004). Molecular detection of prostate cancer: a role for GSTP1 hypermethylation. European urology.

[182]  Meiers I, Shanks JH, Bostwick DG (2007). Glutathione S-transferase pi (GSTP1) hypermethylation in prostate cancer: review 2007. Pathology.

[183]  Perry AS, Foley R, Woodson K, Lawler M (2006). The emerging roles of DNA methylation in the clinical management of prostate cancer. Endocrine-related cancer.

[184]  Li L-C, Okino ST, Dahiya R (2004). DNA methylation in prostate cancer. Biochimica et biophysica acta.

[185]  Antonarakis ES, Carducci M a, Eisenberger M a (2010). Novel targeted therapeutics for metastatic castration-resistant prostate cancer. Cancer letters.

[186]  Zhang Z, Karam J, Frenkel E, Sagalowsky A, Hsieh J-T (2006). The application of epigenetic modifiers on the treatment of prostate and bladder cancer. Urologic Oncol.

[187]  Koeneman KS (2006). Prostate cancer stem cells, telomerase biology, epigenetic modifiers, and molecular systemic therapy for the an drogen-independent lethal phenotype. Urologic Oncol.

[188]  Nelson WG, De Marzo AM, Yegnasubramanian S (2009). Epigenetic alterations in human prostate cancers. Endocrinology.

[189]  Schulz W a, Hoffmann MJ (2009). Epigenetic mechanisms in the biology of prostate cancer. Seminars in cancer Biol.

[190]  Cooper CS, Foster CS (2009). Concepts of epigenetics in prostate cancer development. British J cancer.

[191]  Piunti A, Pasini D (2011). Epigenetic factors in cancer development: polycomb group proteins. Future oncology (London, England).

[192]  Ahmed H (2010). Promoter Methylation in Prostate Cancer and its Application for the Early Detection of Prostate Cancer Using Serum and Urine Samples. Biomarkers in Cancer.

[193]  Jerónimo C, Esteller M (2010). DNA methylation markers for prostate cancer with a stem cell twist. Cancer prevention research (Philadelphia, Pa.).

[194]  Wang LG, Chiao JW (2010). Prostate cancer chemopreventive activity of phenethyl isothiocyanate through epigenetic regulation (review). International J Oncol.

[195]  Donkena KV, Young CYF, Tindall DJ (2010). Oxidative stress and DNA methylation in prostate cancer. Obstetrics and gynecology international.

[196]  Sun W-jian, Zhou X, Zheng J-hang (2012). Histone acetyltransferases and deacetylases: molecular and clinical implications to gastrointestinal carcinogenesis. Acta biochimica et biophysica Sinica.

[197]  Niwa T, Ushijima T (2010). Induction of epigenetic alterations by chronic inflammation and its significance on carcinogenesis.

[198]  Selaru FM, David S, Meltzer SJ, Hamilton JP (2009). Epigenetic events in gastrointestinal cancer. The Ame J gastroenterology.

[199]  Izzo JG, Ajani J a (2007). Thinking in and out of the box when it comes to gastric cancer and cyclooxygenase-2. J clinical oncology: official J Am Soc Clin Oncoly.

[200]  Tamura G (2006). Alterations of tumor suppressor and tumor-related genes in the development and progression of gastric cancer. World J gastroenterology: WJG.

[201]  Choi IS, Wu TT (2005). Epigenetic alterations in gastric carcinogenesis. Cell Res.

[202]  Tamura G (2002). Genetic and epigenetic alterations of tumor suppressor and tumor-related genes in gastric cancer. Histology and histopathology.

[203]  Fukayama M, Hino R, Uozaki H (2008). Epstein-Barr virus and gastric carcinoma: virus-host interactions leading to carcinoma. Cancer science.

[204]  Uozaki H, Fukayama M (2008). Epstein-Barr virus and gastric carcinoma--viral carcinogenesis through epigenetic mechanisms. International J clinical and experimental pathology.

[205]  Li HP, Leu YW, Chang YS (2005). Epigenetic changes in virus-associated human cancers. Cell Res.

[206]  Pero R, Peluso S, Angrisano T (2011). Chromatin and DNA methylation dynamics of Helicobacter pylori-induced COX-2 activation. International J medical microbiology: IJMM.

[207]  Farinati F, Cardin R, Cassaro M (2008). Helicobacter pylori inflammation oxidative damage and gastric cancer: a morphological, biological and molecular pathway. Eur J cancer prevention: the official J Eur Cancer Prevention Organisation (ECP).

[208]  Ree AH, Dueland S, Folkvord S (2010). Vorinostat, a histone deacetylase inhibitor, combined with pelvic palliative radiotherapy for gastrointestinal carcinoma: the Pelvic Radiation and Vorinostat (PRAVO) phase 1 study. The lancet Oncol.

[209]  Shukla SD, Aroor AR (2006). Epigenetic effects of ethanol on liver and gastrointestinal injury. World J gastroenterology: WJG.

[210]  Herceg Z, Paliwal A (2011). Epigenetic mechanisms in hepatocellular
carcinoma: how environmental factors influence the epigenome. Mutation Res.

[211]  Kew MC (2011). Hepatitis B virus x protein in the pathogenesis of hepatitis
B virus-induced hepatocellular carcinoma. J gastroenterology and hepatology.

[212]  Herceg Z, Paliwal A (2009). HBV protein as a double-barrel shot-gun
targets epigenetic landscape in liver cancer. J hepatology.

[213]  Ozturk M, Arslan-Ergul A, Bagislar S, Senturk S, Yuzugullu H (2009). Senescence and immortality in hepatocellular carcinoma. Cancer letters.

[214]  Herath NI, Leggett B a, MacDonald G a (2006). Review of genetic and
epigenetic alterations in hepatocarcinogenesis. J gastroenterology and hepatology.

[215]  Tischoff I (2008). DNA methylation in hepatocellular carcinoma. World J Gastroenterology.

[216]  Issa J-pierre (2002). Epigenetic variation and human disease. J nutrition.

[217]  Lachenmayer A, Alsinet C, Chang CY, Llovet JM (2010). Molecular
approaches to treatment of hepatocellular carcinoma. Digestive and
liver disease: official J Italian Society of Gastroenterology and the
Italian Association for the Study of the Liver.

[218]  Rivenbark AG, Coleman WB (2007). The use of epigenetic biomarkers for
preclinical detection of hepatocellular carcinoma: potential for noninvasive
screening of high-risk populations. Clinical cancer research : an official J Am Ass Cancer Res.

[219]  Venturelli S, Armeanu S, Pathil A (2007). Epigenetic combination
therapy as a tumor-selective treatment approach for hepatocellular
carcinoma. Cancer.

[220]  Lai J-P, Yu C, Moser CD (2006). SULF1 inhibits tumor growth and
potentiates the effects of histone deacetylase inhibitors in hepatocellular
carcinoma. Gastroenterology.

[221]  Coradini D, Speranza A (2005). Invited review Histone deacetylase inhibitors for treatment of hepatocellular carcinoma.

[222]  Arai E, Kanai Y (2010). Genetic and epigenetic alterations during renal carcinogenesis. International J clinical and experimental pathology.

[223]  Dressler GR (2008). Epigenetics, development, and the kidney. J American
Society of Nephrology: JASN.

[224]  Gan HK, Seruga B, Knox JJ (2009). Targeted Therapies for Renal Cell
Carcinoma - More Gains from Using Them Again. Curr Oncol.

[225]  Cang S, Ma Y, Liu D (2009). New clinical developments in histone deacetylase
inhibitors for epigenetic therapy of cancer. J Hematol &Oncol.

[226]  Mai A, Cheng D, Bedford MT (2008). Epigenetic multiple ligands:
mixed histone/protein methyltransferase, acetyltransferase, and
class III deacetylase (sirtuin) inhibitors. J medicinal Chem.

[227]  Medina-Franco JL, Caulfield T (2011). Advances in the computational
development of DNA methyltransferase inhibitors. Drug Discov Today.

[228]  Mani S, Herceg Z (2010). DNA demethylating agents and epigenetic therapy of cancer.

[229]  Karberg S (2009). Switching on epigenetic therapy. Cell.

[230]  Hamm C a, Costa FF (2011). The impact of epigenomics on future drug design and new therapies. Drug Discov Today.

[231]  Sippl W, Jung M (2011). Epigenetic drug discovery special issue. Bioorganic Med Chem.

[232]  Ropero S, Esteller M (2007). The role of histone deacetylases (HDACs) in human cancer. Molecular Oncol.

[233]  Donepudi S, Mattison RJ, Kihslinger JE, Godley L a (2007). Modulators of DNA methylation and histone acetylation. Update on Cancer Therapeutics.

[234]  Iglesias-Linares a, Yañez-Vico RM, González-Moles M a (2010). Potential role of HDAC inhibitors in cancer therapy: insights into oral squamous cell carcinoma. Oral Oncol.

[235]  Hildmann C, Riester D, Schwienhorst A (2007). Histone deacetylases--an important class of cellular regulators with a variety of functions. Applied microbiology and biotechnology.

[236]  Mai A, Massa S, Rotili D (2005). Histone deacetylation in epigenetics: an attractive target for anticancer therapy. Medicinal research reviews.

[237]  Pan LN, Lu J, Huang B (2007). HDAC inhibitors: a potential new category of anti-tumor agents. Cellular &molecular immunology.

[238]  Rajendran P, Williams DE, Ho E, Dashwood RH (2011). Metabolism as a
key to histone deacetylase inhibition. Critical reviews in biochemistry
and molecular Biol.

[239]  Finnin MS, Donigian JR, Cohen A (1999). Structures of a histone
deacetylase homologue bound to the TSA and SAHA inhibitors. Nature.

[240]  Di Marcotullio L, Canettieri G, Infante P, Greco A, Gulino A (2011). Protected from the inside: endogenous histone deacetylase inhibitors
and the road to cancer. Biochimica et biophysica acta.

[241]  Marsoni S, Damia G, Camboni G (2008). A work in progress: the clinical
development of histone deacetylase inhibitors. Epigenetics: official
J DNA Methylation Society.

[242]  Monneret C (2005). Histone deacetylase inhibitors. Eur J medicinal Chem.

[243]  Cea M, Cagnetta A, Gobbi M (2013). New Insights into the Treatment
of Multiple Myeloma with Histone Deacetylase Inhibitors. Curr Pharm Des.

[244]  Dowling DP, Gattis SG, Fierke C a, Christianson DW (2010). Structures
of Metal-Substituted Human Histone Deacetylase 8 Provide
Mechanistic Inferences on Biological Function. BioChem.

[245]  Gantt SL, Gattis SG, Fierke C a (2006). Catalytic activity and inhibition of human histone deacetylase 8 is dependent on the identity of the active site metal ion. BioChem.

[246]  Lombardi PM, Cole KE, Dowling DP, Christianson DW (2011). Structure mechanism and inhibition of histone deacetylases and related metalloenzymes. Curr Opinion Structural Biol.

[247]  Bressi JC, Jennings AJ, Skene R (2010). Exploration of the HDAC2
foot pocket: Synthesis and SAR of substituted N-(2-
aminophenyl)benzamides. Bioorganic Med Chem letters.

[248]  Watson PJ, Fairall L, Santos GM, Schwabe JWR (2012). Structure of
HDAC3 bound to co-repressor and inositol tetraphosphate. Nature.

[249]  Somoza JR, Skene RJ, Katz B a (2004). Structural snapshots of human
HDAC8 provide insights into the class I histone deacetylases. Structure (London, England: 1993).

[250]  Vannini A, Volpari C, Filocamo G (2004). Crystal structure of a
eukaryotic zinc-dependent histone deacetylase human HDAC8,
complexed with a hydroxamic acid inhibitor. Proc Nat Acad Sci
USA.

[251]  Vannini A, Volpari C, Gallinari P (2007). Substrate binding to histone
deacetylases as shown by the crystal structure of the HDAC8-
substrate complex. EMBO reports.

[252]  Dowling DP, Gantt SL, Gattis SG, Fierke C a, Christianson DW (2008). Structural studies of human histone deacetylase 8 and its sitespecific
variants complexed with substrate and inhibitors. BioChem.

[253]  Cole KE, Dowling DP, Boone MA, Phillips AJ, Christianson DW (2011). Structural basis of the antiproliferative activity of largazole, a depsipeptide
inhibitor of the histone deacetylases. J American Chemical Society.

[254]  Whitehead L, Dobler MR, Radetich B (2011). Human HDAC isoform
selectivity achieved via exploitation of the acetate release
channel with structurally unique small molecule inhibitors. Bioorganic Med Chem.

[255]  Guo L, Han A, Bates DL, Cao J, Chen L (2007). Crystal structure of a
conserved N-terminal domain of histone deacetylase 4 reveals
functional insights into glutamine-rich domains. Proc Nat Acad Sci USA.

[256]  Bottomley MJ, Lo Surdo P, Di Giovine P (2008). Structural and
functional analysis of the human HDAC4 catalytic domain reveals
a regulatory structural zinc-binding domain. J Biol Chem.

[257]  Schuetz A, Min J, Allali-Hassani A (2008). Human HDAC7 harbors
a class IIa histone deacetylase-specific zinc binding motif and cryptic
deacetylase activity. J Biol Chem.

[258]  Ouyang H, Ali YO, Ravichandran M (2012). Protein aggregates are
recruited to aggresome by histone deacetylase 6 via unanchored
ubiquitin C termini. J Biol Chem.

[259]  Nielsen TK, Hildmann C, Dickmanns A, Schwienhorst A, Ficner 
R (2005). Crystal structure of a bacterial class 2 histone deacetylase homologue. J Mol Biolo.

[260]  Nielsen TK, Hildmann C, Riester D, Wegener D, Schwienhorst A, 
Ficner R (2007). Complex structure of a bacterial class 2 histone deacetylase
homologue with a trifluoromethylketone inhibitor. Acta crystallographica.
Section F, Structural biology and crystallization
communications.

[261]  Schäfer S, Saunders L, Eliseeva E (2008). Phenylalanine-containing
hydroxamic acids as selective inhibitors of class IIb histone deacetylases
(HDACs). Bioorganic Med Chem.

[262]  Lombardi PM, Angell HD, Whittington D a, Flynn EF, Rajashankar 
KR, Christianson DW (2011). Structure of prokaryotic polyamine
deacetylase reveals evolutionary functional relationships
with eukaryotic histone deacetylases. BioChem.

[263]  Abendroth J, Gardberg AS, Robinson JI (2011). SAD phasing using
iodide ions in a high-throughput structural genomics environment. J structural and functional genomics.

[264]  Brachmann CB, Sherman JM, Devine SE, Cameron EE, Pillus L, 
Boeke JD (1995). The SIR2 gene family, conserved from bacteria to humans,
functions in silencing, cell cycle progression, and chromosome
stability. Genes &development.

[265]  Libert S, Pointer K, Bell EL (2011). SIRT1 activates MAO-A in the
brain to mediate anxiety and exploratory drive. Cell.

[266]  Finkel T, Deng C-X, Mostoslavsky R (2009). Recent progress in the biology
and physiology of sirtuins. Nature.

[267]  Min J, Landry J, Sternglanz R, Xu RM (2001). Crystal structure of a SIR2 homolog-NAD complex. Cell.

[268]  Avalos JL, Bever KM, Wolberger C (2005). Mechanism of sirtuin inhibition
by nicotinamide: altering the NAD(+) cosubstrate specificity
of a Sir2 enzyme. Molecular cell.

[269]  Sanders BD, Zhao K, Slama JT, Marmorstein R (2007). Structural basis
for nicotinamide inhibition and base exchange in Sir2 enzymes. Molecular cell.

[270]  Schuetz A, Min J, Antoshenko T (2007). Structural basis of inhibition
of the human NAD+-dependent deacetylase SIRT5 by
suramin. Structure (London, England: 1993).

[271]  Bruzzone S, Parenti MD, Grozio A, Bauer I, Del Rio A, Nencioni 
A (2013). Rejuvenating sirtuins: the rise of a new family of cancer drug
targets. Curr Pharm Des.

[272]  Finnin MS, Donigian JR, Pavletich NP (2001). Structure of the histone
deacetylase SIRT2. Nature structural Biol.

[273]  Jin L, Wei W, Jiang Y (2009). Crystal structures of human SIRT3
displaying substrate-induced conformational changes. J Biol Chem.

[274]  Du J, Zhou Y, Su X (2011). Sirt5 is a NAD-dependent protein lysine
demalonylase and desuccinylase. Science (New York, N.Y.).

[275]  Pan PW, Feldman JL, Devries MK, Dong A, Edwards AM, Denu 
JM (2011). Structure and biochemical functions of SIRT6. J Biol Chem.

[276]  Cosgrove MS, Bever K, Avalos JL, Muhammad S, Zhang X, Wolberger 
C (2006). The structural basis of sirtuin substrate affinity. BioChem.

[277]  Hoff KG, Avalos JL, Sens K, Wolberger C (2006). Insights into the sirtuin
mechanism from ternary complexes containing NAD+ and acetylated
peptide. Structure (London, England: 1993).

[278]  Hawse WF, Hoff KG, Fatkins DG (2008). Structural insights into
intermediate steps in the Sir2 deacetylation reaction. Structure
(London, England: 1993).

[279]  Hawse WF, Wolberger C (2009). Structure-based mechanism of ADPribosylation
by sirtuins. J Biol Chem.

[280]  Bheda P, Wang JT, Escalante-Semerena JC, Wolberger C (2011). Structure
of Sir2Tm bound to a propionylated peptide. Protein science: a
publication of the Protein Society.

[281]  Avalos JL, Boeke JD, Wolberger C (2004). Structural basis for the mechanism
and regulation of Sir2 enzymes. Molecular cell.

[282]  Avalos JL, Celic I, Muhammad S, Cosgrove MS, Boeke JD, Wolberger 
C (2002). Structure of a Sir2 enzyme bound to an acetylated p53 peptide. Molecular cell.

[283]  Zhao K, Chai X, Clements A, Marmorstein R (2003). Structure and
autoregulation of the yeast Hst2 homolog of Sir2. Nature structural
Biol.

[284]  Zhao K, Chai X, Marmorstein R (2003). Structure of the Yeast Hst2 Protein
Deacetylase in Ternary Complex with 2??-O-Acetyl ADP Ribose
and Histone Peptide. Structure.

[285]  Zhao K, Harshaw R, Chai X, Marmorstein R (2004). Structural basis for
nicotinamide cleavage and ADP-ribose transfer by NAD(+)-
dependent Sir2 histone/protein deacetylases. Proc Nat Acad Sci
USA.

[286]  Chang JH, Kim HC, Hwang KY (2002). Structural basis for the
NAD-dependent deacetylase mechanism of Sir2. J Biol Chem.

[287]  Zhu AY, Zhou Y, Khan S, Deitsch KW, Hao Q, Lin H (2012). Plasmodium
falciparum Sir2A preferentially hydrolyzes medium and long
chain fatty acyl lysine. ACS chemical Biol.

[288]  Hodawadekar SC, Marmorstein R (2007). Chemistry of acetyl transfer by
histone modifying enzymes: structure, mechanism and implications
for effector design. Oncogene.

[289]  Parthun MR (2007). Hat1: the emerging cellular roles of a type B histone
acetyltransferase. Oncogene.

[290]  Marmorstein R (2001). Structure of histone acetyltransferases. J Mol Biolo.

[291]  Lee KK, Workman JL (2007). Histone acetyltransferase complexes: one
size doesn’t fit all. Nat Rev Molecular cell Biol.

[292]  Hudson BP, Martinez-Yamout M a, Dyson HJ, Wright PE (2000). Solution
structure and acetyl-lysine binding activity of the GCN5 bromodomain. J Mol Biolo.

[293]  Schuetz A, Bernstein G, Dong A (2007). Crystal structure of a binary
complex between human GCN5 histone acetyltransferase domain
and acetyl coenzyme A. Proteins.

[294]  Filippakopoulos P, Picaud S, Mangos M (2012). Histone recognition
and large-scale structural analysis of the human bromodomain family. Cell.

[295]  Owen DJ, Ornaghi P, Yang JC (2000). The structural basis for the
recognition of acetylated histone H4 by the bromodomain of histone
acetyltransferase gcn5p. EMBO J.

[296]  Trievel RC, Rojas JR, Sterner DE (1999). Crystal structure and
mechanism of histone acetylation of the yeast GCN5 transcriptional
coactivator. Proc Nat Acad Sci USA.

[297]  Clements a, Rojas JR, Trievel RC, Wang L, Berger SL, Marmorstein 
R (1999). Crystal structure of the histone acetyltransferase domain of
the human PCAF transcriptional regulator bound to coenzyme A. EMBO journal.

[298]  Mujtaba S, He Y, Zeng L (2002). Structural basis of lysineacetylated
HIV-1 Tat recognition by PCAF bromodomain. Molecular cell.

[299]  Dhalluin C, Carlson JE, Zeng L, He C, Aggarwal AK, Zhou MM (1999). Structure and ligand of a histone acetyltransferase bromodomain. Nature.

[300]  Zeng L, Li J, Muller M (2005). Selective small molecules blocking
HIV-1 Tat and coactivator PCAF association. J American Chemical Society.

[301]  Zeng L, Zhang Q, Gerona-Navarro G, Moshkina N, Zhou MM (2008). Structural basis of site-specific histone recognition by the bromodomains
of human coactivators PCAF and CBP/p300. Structure (London, England: 1993).

[302]  Angus-Hill ML, Dutnall RN, Tafrov ST, Sternglanz R, Ramakrishnan V (1999). Crystal structure of the histone acetyltransferase Hpa2: A
tetrameric member of the Gcn5-related N-acetyltransferase superfamily. J Mol Biolo.

[303]  Freedman SJ, Sun Z-YJ, Poy F (2002). Structural basis for recruitment
of CBP/p300 by hypoxia-inducible factor-1 alpha. Proc Nat
Acad Sci USA.

[304]  Freedman SJ, Sun Z-yu J, Kung AL, France DS, Wagner G, Eck MJ (2003). Structural basis for negative regulation of hypoxia-inducible
factor-1alpha by CITED2. Nature structural Biol.

[305]  Feng H, Jenkins LMM, Durell SR (2009). Structural basis for p300
Taz2-p53 TAD1 binding and modulation by phosphorylation. Structure (London, England: 1993).

[306]  Liu X, Wang L, Zhao K (2008). The structural basis of protein acetylation
by the p300/CBP transcriptional coactivator. Nature.

[307]  Miller M, Dauter Z, Cherry S, Tropea JE, Wlodawer A (2009). Structure
of the Taz2 domain of p300: insights into ligand binding. Acta
crystallographica. Section D, Biological crystallography.

[308]  He J, Ye J, Cai Y (2011). Structure of p300 bound to MEF2 on DNA
reveals a mechanism of enhanceosome assembly. Nucleic acids Res.

[309]  Mujtaba S, He Y, Zeng L (2004). Structural mechanism of the bromodomain
of the coactivator CBP in p53 transcriptional activation. Molecular cell.

[310]  Sharpe BK, Matthews JM, Kwan AHY (2002). A new zinc binding
fold underlines the versatility of zinc binding modules in protein
evolution. Structure (London England: 1993).

[311]  Sharpe BK, Liew CK, Kwan AH (2005). Assessment of the robustness
of a serendipitous zinc binding fold: mutagenesis and protein
grafting. Structure (London, England: 1993).

[312]  Hiscott J, Lin R (2005). IRF-3 releases its inhibitions. Structure (London, England: 1993).

[313]  Sachchidanand Resnick-Silverman L, Yan S (2006). Target structure-
based discovery of small molecules that block human p53 and
CREB binding protein association. Chemistry &Biol.

[314]  Ferreon JC, Martinez-Yamout M a, Dyson HJ, Wright PE (2009). Structural
basis for subversion of cellular control mechanisms by the
adenoviral E1A oncoprotein. Proc Nat Acad Sci USA.

[315]  Borah JC, Mujtaba S, Karakikes I (2011). A small molecule binding
to the coactivator CREB-binding protein blocks apoptosis in cardiomyocytes. Chemistry &Biol.

[316]  Chung C-W, Dean AW, Woolven JM, Bamborough P (2012). Fragmentbased
discovery of bromodomain inhibitors part 1: inhibitor binding
modes and implications for lead discovery. J medicinal Chem.

[317]  De Guzman RN, Liu HY, Martinez-Yamout M, Dyson HJ, Wright 
PE (2000). Solution structure of the TAZ2 (CH3) domain of the transcriptional
adaptor protein CBP. J Mol Biolo.

[318]  Lin CH, Hare BJ, Wagner G, Harrison SC, Maniatis T, Fraenkel E (2001). A small domain of CBP/p300 binds diverse proteins: solution
structure and functional studies. Molecular cell.

[319]  Demarest SJ, Martinez-Yamout M, Chung J (2002). Mutual synergistic
folding in recruitment of CBP/p300 by p160 nuclear receptor
coactivators. Nature.

[320]  Radhakrishnan I, Pérez-Alvarado GC, Parker D, Dyson HJ, Montminy MR, Wright PE (1997). Solution structure of the KIX domain of
CBP bound to the transactivation domain of CREB: a model for activator:
coactivator interactions. Cell.

[321]  Dames S a, Martinez-Yamout M, De Guzman RN, Dyson HJ, Wright PE (2002). Structural basis for Hif-1 alpha /CBP recognition in the
cellular hypoxic response. Proc Nat Acad Sci USA.

[322]  De Guzman RN, Martinez-Yamout M a, Dyson HJ, Wright PE (2004). Interaction of the TAZ1 domain of the CREB-binding protein with
the activation domain of CITED2: regulation by competition between
intrinsically unstructured ligands for non-identical binding
sites. J Biol Chem.

[323]  Zor T, De Guzman RN, Dyson HJ, Wright PE (2004). Solution structure
of the KIX domain of CBP bound to the transactivation domain of
c-Myb. J Mol Biolo.

[324]  Legge. GB. Martinez-Yamout M a, Hambly  DM (2004). ZZ domain
of CBP: an unusual zinc finger fold in a protein interaction module. J Mol Biolo.

[325]  De Guzman RN, Wojciak JM, Martinez-Yamout M a, Dyson HJ, Wright PE (2005). CBP/p300 TAZ1 domain forms a structured scaffold for ligand binding. BioChem.

[326]  De Guzman RN, Goto NK, Dyson HJ, Wright PE (2006). Structural basis
for cooperative transcription factor binding to the CBP coactivator. J Mol Biolo.

[327]  Waters L, Yue B, Veverka V (2006). Structural diversity in
p160/CREB-binding protein coactivator complexes. J Biol Chem.

[328]  Wojciak JM, Martinez-Yamout M a, Dyson HJ, Wright PE (2009). Structural
basis for recruitment of CBP/p300 coactivators by STAT1 and
STAT2 transactivation domains. EMBO J.

[329]  Kjaergaard M, Teilum K, Poulsen FM (2010). Conformational selection in
the molten globule state of the nuclear coactivator binding domain
of CBP. Proc Nat Acad Sci USA.

[330]  Lee CW, Martinez-Yamout M a, Dyson HJ, Wright PE (2010). Structure
of the p53 transactivation domain in complex with the nuclear receptor
coactivator binding domain of CREB binding protein. Bio-Chem.

[331]  Holbert M a, Sikorski T, Carten J, Snowflack D, Hodawadekar S, 
Marmorstein R (2007). The human monocytic leukemia zinc finger histone
acetyltransferase domain contains DNA-binding activity im608
plicated in chromatin targeting. J Biol Chem.

[332]  Kadlec J, Hallacli E, Lipp M (2011). Structural basis for MOF and
MSL3 recruitment into the dosage compensation complex by
MSL1. Nature structural Mol Biol.

[333]  Sun B, Guo S, Tang Q (2011). Regulation of the histone acetyltransferase
activity of hMOF via autoacetylation of Lys274. Cell Res.

[334]  Yuan H, Rossetto D, Mellert H (2012). MYST protein acetyltransferase
activity requires active site lysine autoacetylation. EMBO J.

[335]  Yan Y, Barlev N a, Haley RH, Berger SL, Marmorstein R (2000). Crystal
structure of yeast Esa1 suggests a unified mechanism for catalysis
and substrate binding by histone acetyltransferases. Molecular cell.

[336]  Yan Y, Harper S, Speicher DW, Marmorstein R (2002). The catalytic
mechanism of the ESA1 histone acetyltransferase involves a selfacetylated
intermediate. Nature structural Biol.

[337]  Shimojo H, Sano N, Moriwaki Y, Okuda M, Horikoshi M, Nishimura Y (2008). Novel structural and functional mode of a knot essential
for RNA binding activity of the Esa1 presumed chromodomain. J Mol Biolo.

[338]  Dutnall RN, Tafrov ST, Sternglanz R, Ramakrishnan V (1998). Structure
of the histone acetyltransferase Hat1: a paradigm for the GCN5-
related N-acetyltransferase superfamily. Cell.

[339]  Lin C, Yuan YA (2008). Structural insights into histone H3 lysine 56
acetylation by Rtt109. Structure (London, England: 1993).

[340]  Stavropoulos P, Nagy V, Blobel G, Hoelz A (2008). Molecular basis for
the autoregulation of the protein acetyl transferase Rtt109. Proc Nat Acad Sci USA.

[341]  Tang Y, Holbert M a, Delgoshaie N (2011). Structure of the Rtt109-
AcCoA/Vps75 complex and implications for chaperone-mediated
histone acetylation. Structure (London, England: 1993).

[342]  Su D, Hu Q, Zhou H (2011). Structure and histone binding properties
of the Vps75-Rtt109 chaperone-lysine acetyltransferase complex. J Biol Chem.

[343]  Tang Y, Holbert MA, Wurtele H (2008). Fungal Rtt109 histone
acetyltransferase is an unexpected structural homolog of metazoan
p300/CBP. Nature structural Mol Biol.

[344]  Chung C-wa, Tough DF (2012). Bromodomains: a new target class for
small molecule drug discovery. Drug Discov Today: Therapeutic
Strategies.

[345]  Loyola A, Almouzni G (2004). Bromodomains in living cells participate in
deciphering the histone code. Trends in cell Biol.

[346]  Jones MH, Hamana N, Nezu JI, Shimane M (2000). A novel family of bromodomain genes. Genomics.

[347]  Filippakopoulos P, Qi J, Picaud S (2010). Selective inhibition of
BET bromodomains. Nature.

[348]  Chung C-W, Coste H, White JH (2011). Discovery and characterization
of small molecule inhibitors of the BET family bromodomains. J medicinal Chem.

[349]  Blobel G a, Kalota A, Sanchez PV, Carroll M (2011). Short hairpin RNA
screen reveals bromodomain proteins as novel targets in acute myeloid
leukemia. Cancer cell.

[350]  Dawson M a, Prinjha RK, Dittmann A (2011). Inhibition of BET
recruitment to chromatin as an effective treatment for MLL-fusion
leukaemia. Nature.

[351]  Hewings DS, Wang M, Philpott M (2011). 3,5-dimethylisoxazoles
act as acetyl-lysine-mimetic bromodomain ligands. J medicinal Chem.

[352]  Bamborough P, Diallo H, Goodacre JD (2012). Fragment-based
discovery of bromodomain inhibitors part 2: optimization of phenylisoxazole
sulfonamides. J medicinal Chem.

[353]  Mertz J a, Conery AR, Bryant BM (2011). Targeting MYC dependence
in cancer by inhibiting BET bromodomains. Proc Nat Acad Sci USA.

[354]  Di Lorenzo A, Bedford MT (2011). Histone arginine methylation. FEBS letters.

[355]  Nimura K, Ura K, Kaneda Y (2010). Histone methyltransferases: regulation
of transcription and contribution to human disease. J molecular
medicine (Berlin, Germany).

[356]  Copeland RA, Richon VM (2011). The human protein methyltransferases.

[357]  Martinet N, Michel BY, Bertrand P, Benhida R (2011). Small molecules
DNA methyltransferases inhibitors. MedChemComm.

[358]  Daniel FI, Cherubini K, Yurgel LS, de Figueiredo MAZ, Salum FG (2011). The role of epigenetic transcription repression and DNA methyltransferases in cancer. Cancer.

[359]  Ferrari KJ, Pasini D (2013). Regulation and Function of DNA and Histone Methylations. Curr Pharm Des.

[360]  Fandy TE (2009). Development of DNA methyltransferase inhibitors for
the treatment of neoplastic diseases. Curr medicinal Chem.

[361]  Yoo J, Medina-Franco JL (2011). Discovery and Optimization of Inhibitors
of DNA Methyltransferase as Novel Drugs for Cancer Therapy.

[362]  Albert M, Helin K (2010). Histone methyltransferases in cancer. Seminars in cell &developmental Biol.

[363]  Burgers W a, Fuks F, Kouzarides T (2002). DNA methyltransferases get connected to chromatin. Trends in genetics: TIG.

[364]  Chang Y, Ganesh T, Horton JR (2010). Adding a lysine mimic in the
design of potent inhibitors of histone lysine methyltransferases. J
Mol Biolo.

[365]  Min J, Feng Q, Li Z, Zhang Y, Xu R-ming (2003). Structure of the catalytic
domain of human DOT1L, a non-SET domain nucleosomal
histone methyltransferase. Cell.

[366]  Richon VM, Johnston D, Sneeringer CJ (2011). Chemogenetic analysis of human protein methyltransferases. Chemical biology &drug design.

[367]  Yao Y, Chen P, Diao J (2011). Selective inhibitors of histone methyltransferase DOT1L: design, synthesis, and crystallographic studies. J American Chemical Society.

[368]  Southall SM, Wong P-S, Odho Z, Roe SM, Wilson JR (2009). Structural
basis for the requirement of additional factors for MLL1 SET domain
activity and recognition of epigenetic marks. Molecular cell.

[369]  Wang Z, Song J, Milne T a (2010). Pro isomerization in MLL1
PHD3-bromo cassette connects H3K4me readout to CyP33 and
HDAC-mediated repression. Cell.

[370]  Park S, Osmers U, Raman G, Schwantes RH, Diaz MO, Bushweller JH (2010). The PHD3 domain of MLL acts as a CYP33-regulated switch between MLL-mediated activation and repression. Bio-Chem.

[371]  Wu H, Min J, Lunin VV (2010). Structural biology of human H3K9 methyltransferases. PloS one.

[372]  Collins RE, Northrop JP, Horton JR (2008). The ankyrin repeats of
G9a and GLP histone methyltransferases are mono- and dimethyllysine
binding modules. Nature structural Mol Biol.

[373]  Chang Y, Zhang X, Horton JR (2009). Structural basis for G9a-like protein lysine methyltransferase inhibition by BIX-01294. Nature structural Mol Biol.

[374]  Chang Y, Sun L, Kokura K (2011). MPP8 mediates the interactions
between DNA methyltransferase Dnmt3a and H3K9 methyltransferase
GLP/G9a. Nature communications.

[375]  Liu F, Chen X, Allali-Hassani A (2009). Discovery of a 2,4-diamino-
7-aminoalkoxyquinazoline as a potent and selective inhibitor of
histone lysine methyltransferase G9a. J medicinal Chem.

[376]  Vedadi M, Barsyte-Lovejoy D, Liu F (2011). A chemical probe
selectively inhibits G9a and GLP methyltransferase activity in
cells. Nature chemical Biol.

[377]  Qiao Q, Li Y, Chen Z, Wang M, Reinberg D, Xu R-M (2011). The structure
of NSD1 reveals an autoregulatory mechanism underlying histone
H3K36 methylation. J Biol Chem.

[378]  Chang Y, Levy D, Horton JR (2011). Structural basis of SETD6-
mediated regulation of the NF-kB network via methyl-lysine signaling. Nucleic acids Res.

[379]  Wilson JR, Jing C, Walker PA (2002). Crystal structure and functional
analysis of the histone methyltransferase SET7/9. Cell.

[380]  Jacobs SA, Harp JM, Devarakonda S,  Kim Y, Rastinejad F, 
Khorasanizadeh S (2002). The active site of the SET domain is constructed on a knot. Nature structural Biol.

[381]  Kwon T, Chang JH, Kwak E (2003). Mechanism of histone lysine
methyl transfer revealed by the structure of SET7/9-AdoMet. EMBO J.

[382]  Xiao B, Jing C, Wilson JR (2003). Structure and catalytic mechanism
of the human histone methyltransferase SET7/9. Nature.

[383]  Chuikov S, Kurash JK, Wilson JR (2004). Regulation of p53 activity
through lysine methylation. Nature.

[384]  Couture J-F, Collazo E, Hauk G, Trievel RC (2006). Structural basis for
the methylation site specificity of SET7/9. Nature structural Mol Biol.

[385]  Subramanian K, Jia D, Kapoor-Vazirani P (2008). Regulation of
estrogen receptor alpha by the SET7 lysine methyltransferase. Molecular cell.

[386]  Estève P-O, Chang Y, Samaranayake M (2011). A methylation and
phosphorylation switch between an adjacent lysine and serine determines
human DNMT1 stability. Nature structural Mol Biol.

[387]  Couture J-F, Collazo E, Brunzelle JS, Trievel RC (2005). Structural and
functional analysis of SET8, a histone H4 Lys-20 methyltransferase. Genes &development.

[388]  Xiao B, Jing C, Kelly G (2005). Specificity and mechanism of the
histone methyltransferase Pr-Set7. Genes &development.

[389]  Couture J-F, Dirk LM a, Brunzelle JS, Houtz RL, Trievel RC (2008). Structural origins for the product specificity of SET domain protein
methyltransferases. Proc Nat Acad Sci USA.

[390]  Li M, Phatnani HP, Guan Z, Sage H, Greenleaf AL, Zhou P (2005). Solution
structure of the Set2-Rpb1 interacting domain of human Set2
and its interaction with the hyperphosphorylated C-terminal domain
of Rpb1. Proc Nat Acad Sci USA.

[391]  Goodwin KD, He H, Imasaki T, Lee S-H, Georgiadis MM (2010). Crystal
structure of the human Hsmar1-derived transposase domain in the
DNA repair enzyme Metnase. BioChem.

[392]  Sirinupong N, Brunzelle J, Ye J, Pirzada A, Nico L, Yang Z (2010). Crystal
structure of cardiac-specific histone methyltransferase SmyD1
reveals unusual active site architecture. J Biol Chem.

[393]  Jiang Y, Sirinupong N, Brunzelle J, Yang Z (2011). Crystal structures of
histone and p53 methyltransferase SmyD2 reveal a conformational
flexibility of the autoinhibitory C-terminal domain. PloS one.

[394]  Xu S, Zhong C, Zhang T, Ding J (2011). Structure of human lysine methyltransferase
Smyd2 reveals insights into the substrate divergence
in Smyd proteins. J molecular cell Biol.

[395]  Ferguson AD, Larsen N a, Howard T (2011). Structural basis of
substrate methylation and inhibition of SMYD2. Structure (London,
England: 1993).

[396]  Wang L, Li L, Zhang H (2011). Structure of human SMYD2 protein
reveals the basis of p53 tumor suppressor methylation. J Biol Chem.

[397]  Xu S, Wu J, Sun B, Zhong C, Ding J (2011). Structural and biochemical
studies of human lysine methyltransferase Smyd3 reveal the important
functional roles of its post-SET and TPR domains and the
regulation of its activity by DNA binding. Nucleic acids Res.

[398]  Sirinupong N, Brunzelle J, Doko E, Yang Z (2011). Structural insights into
the autoinhibition and posttranslational activation of histone methyltransferase
SmyD3. J Mol Biolo.

[399]  Foreman KW, Brown M, Park F (2011). Structural and functional
profiling of the human histone methyltransferase SMYD3. PloS one.

[400]  Briknarová K, Zhou X, Satterthwait A, Hoyt DW, Ely KR, Huang S (2008). Structural studies of the SET domain from RIZ1 tumor suppressor. Biochemical and biophysical research communications.

[401]  Briknarová K, Atwater DZ, Glicken JM, Maynard SJ, Ness TE (2011). The PR/SET domain in PRDM4 is preceded by a zinc knuckle. Proteins.

[402]  Troffer-Charlier N, Cura V, Hassenboehler P, Moras D, Cavarelli J (2007). Functional insights from structures of coactivator-associated arginine
methyltransferase 1 domains. EMBO J.

[403]  Sack JS, Thieffine S, Bandiera T (2011). Structural basis for
CARM1 inhibition by indole and pyrazole inhibitors. Biochem J.

[404]  Yue WW, Hassler M,  Roe SM, Thompson-Vale V, Pearl LH (2007). Insights
into histone code syntax from structural and biochemical
studies of CARM1 methyltransferase. EMBO J.

[405]  Zhang X, Cheng X (2003). Structure of the Predominant Protein Arginine
Methyltransferase PRMT1 and Analysis of Its Binding to Substrate
Peptides. Structure.

[406]  Zhang X, Zhou L, Cheng X (2000). Crystal structure of the conserved core
of protein arginine methyltransferase PRMT3. EMBO J.

[407]  Tempel W, Wu H, Dombrovsky L (2009). An intact SAM-dependent
methyltransferase fold is encoded by the human endothelinconverting
enzyme-2 gene. Proteins.

[408]  Cheng. X/ Blumenthal RM (2008). Mammalian DNA methyltransferases: a
structural perspective. Structure (London, England: 1993).

[409]  Chen Z-xia, Riggs AD (2011). DNA methylation and demethylation in mammals. J Biol Chem.

[410]  Bestor TH (2000). The DNA methyltransferases of mammals. Human molecular genetics.

[411]  Jurkowska RZ, Jurkowski TP, Jeltsch A (2011). Structure and function of
mammalian DNA methyltransferases. Chembiochem: Eur J chemical Biol.

[412]  Dong a, Yoder J a, Zhang X, Zhou L, Bestor TH, Cheng X (2001). Structure
of human DNMT2, an enigmatic DNA methyltransferase homolog
that displays denaturant-resistant binding to DNA. Nucleic acids Res.

[413]  Yoo J, Medina-Franco JL (2011). Homology modeling, docking and structure-
based pharmacophore of inhibitors of DNA methyltransferase. J computer-aided molecular design.

[414]  Song J, Rechkoblit O, Bestor TH, Patel DJ (2011). Structure of DNMT1-DNA complex reveals a role for autoinhibition in maintenance
DNA methylation. Science (New York, N.Y.).

[415]  Syeda F, Fagan RL, Wean M (2011). The replication focus targeting
sequence (RFTS) domain is a DNA-competitive inhibitor of
Dnmt1. J Biol Chem.

[416]  Jia D, Jurkowska RZ, Zhang X, Jeltsch A, Cheng X (2007). Structure of
Dnmt3a bound to Dnmt3L suggests a model for de novo DNA
methylation. Nature.

[417]  Otani J, Nankumo T, Arita K, Inamoto S, Ariyoshi M, Shirakawa M (2009). Structural basis for recognition of H3K4 methylation status by
the DNA methyltransferase 3A ATRX-DNMT3-DNMT3L domain. EMBO reports.

[418]  Wu H, Zeng H, Lam R (2011). Structural and histone binding ability
characterizations of human PWWP domains. PloS one.

[419]  Qiu C, Sawada K, Zhang X, Cheng X (2002). The PWWP domain of
mammalian DNA methyltransferase Dnmt3b defines a new family
of DNA-binding folds. Nature structural Biol.

[420]  Ooi SKT, Qiu C, Bernstein E (2007). DNMT3L connects unmethylated
lysine 4 of histone H3 to de novo methylation of DNA. Nature.

[421]  Shi Y, Lan F, Matson C (2004). Histone demethylation mediated by
the nuclear amine oxidase homolog LSD1. Cell.

[422]  Tsukada Y-ichi, Fang J, Erdjument-Bromage H (2006). Histone
demethylation by a family of JmjC domain-containing proteins. Nature.

[423]  Kooistra SM, Helin K (2012). Molecular mechanisms and potential functions
of histone demethylases. Nat Rev Molecular Cell Biol.

[424]  Hou H, Yu H (2010). Structural insights into histone lysine demethylation. Curr Opinion Structural Biol.

[425]  Lee MG, Wynder C, Cooch N, Shiekhattar R (2005). An essential role for
CoREST in nucleosomal histone 3 lysine 4 demethylation. Nature.

[426]  Shi Y-J, Matson C, Lan F, Iwase S, Baba T, Shi Y (2005). Regulation of
LSD1 histone demethylase activity by its associated factors. Mol cell.

[427]  Natoli G, Testa G, De Santa F (2009). The future therapeutic potential of
histone demethylases: A critical analysis. Curr opinion in drug discovery &development.

[428]  Lora JM, Wilson DM, Lee K, Larminie CGC (2010). Epigenetic control of
the immune system: histone demethylation as a target for drug discovery. Drug Discov Today: Technologies.

[429]  Varier R a (2011). Timmers HTM. Histone lysine methylation and demethylation
pathways in cancer. Biochimica et biophysica acta.

[430]  Lohse B, Kristensen JL, Kristensen LH (2011). Inhibitors of histone
demethylases. Bioorganic Med Chem.

[431]  Heightman TD (2011). Chemical biology of lysine demethylases. Curr chemical genomics.

[432]  Tochio N, Umehara T, Koshiba S (2006). Solution structure of the
SWIRM domain of human histone demethylase LSD1. Structure (London, England: 1993).

[433]  Mimasu S, Sengoku T, Fukuzawa S, Umehara T, Yokoyama S (2008). Crystal structure of histone demethylase LSD1 and tranylcypromine
at 2.25 A. Biochemical and biophysical research communications.

[434]  Stavropoulos P, Blobel G, Hoelz A (2006). Crystal structure and mechanism
of human lysine-specific demethylase-1. Nature structural
Mol Biol.

[435]  Chen Y, Yang Y, Wang F (2006). Crystal structure of human histone
lysine-specific demethylase 1 (LSD1). Proc Nat Acad Sci USA.

[436]  Yang M, Gocke CB, Luo X (2006). Structural basis for CoRESTdependent
demethylation of nucleosomes by the human LSD1 histone
demethylase. Molecular cell.

[437]  Yang M, Culhane JC, Szewczuk LM (2007). Structural basis of
histone demethylation by LSD1 revealed by suicide inactivation. Nature structural Mol Biol.

[438]  Forneris F, Binda C, Adamo A, Battaglioli E, Mattevi A (2007). Structural
basis of LSD1-CoREST selectivity in histone H3 recognition. J
Biol Chem.

[439]  Zibetti C, Adamo a, Binda C (2010). Alternative Splicing of the
Histone Demethylase LSD1/KDM1 Contributes to the Modulation
of Neurite Morphogenesis in the Mammalian Nervous System. J
Neuroscience.

[440]  Binda C, Valente S, Romanenghi M (2010). Biochemical, structural,
and biological evaluation of tranylcypromine derivatives as inhibitors
of histone demethylases LSD1 and LSD2. J American Chemical
Society.

[441]  Baron R, Binda C, Tortorici M, McCammon JA, Mattevi A (2011). Molecular
mimicry and ligand recognition in binding and catalysis by
the histone demethylase LSD1-CoREST complex. Structure (London,
England: 1993).

[442]  Mimasu S, Umezawa N, Sato S, Higuchi T, Umehara T, Yokoyama 
S (2010). Structurally designed trans-2-phenylcyclopropylamine derivatives
potently inhibit histone demethylase LSD1/KDM1. BioChem.

[443]  Mantri M, Krojer T, Bagg  Ea (2010). Crystal Structure of the 2-
Oxoglutarate- and Fe(II)-Dependent Lysyl Hydroxylase JMJD6. J Mol Biolo.

[444]  Horton JR, Upadhyay AK, Qi HH, Zhang X, Shi Y, Cheng X (2010). Enzymatic and structural insights for substrate specificity of a family
of jumonji histone lysine demethylases. Nature structural Mol
Biol.

[445]  Upadhyay AK, Rotili D, Han JW (2012). An analog of BIX-01294
selectively inhibits a family of histone H3 lysine 9 Jumonji demethylases. J Mol Biolo.

[446]  Yang Y, Hu L, Wang P (2010). Structural insights into a dualspecificity
histone demethylase ceKDM7A from Caenorhabditis
elegans. Cell Res.

[447]  Xu W, Yang H, Liu Y (2011). Oncometabolite 2-hydroxyglutarate is a competitive inhibitor of -ketoglutarate-dependent dioxygenases. Cancer cell.

[448]  Yue WW, Hozjan V, Ge W (2010). Crystal structure of the PHF8
Jumonji domain, an Nepsilon-methyl lysine demethylase. FEBS letters.

[449]  Yu L, Wang Y, Huang S (2010). Structural insights into a novel histone demethylase PHF8. Cell Res.

[450]  Wen H, Li J, Song T (2010). Recognition of histone H3K4 trimethylation
by the plant homeodomain of PHF2 modulates histone demethylation. J Biol Chem.

[451]  Horton JR, Upadhyay AK, Hashimoto H, Zhang X, Cheng X (2011). Structural basis for human PHF2 Jumonji domain interaction with
metal ions. J Mol Biolo.

[452]  Sengoku T, Yokoyama S (2011). Structural basis for histone H3 Lys 27
demethylation by UTX/KDM6A. Genes &development.

[453]  Huang Y, Fang J, Bedford MT, Zhang Y, Xu R-M (2006). Recognition of
histone H3 lysine-4 methylation by the double tudor domain of
JMJD2A. Science (New York, N.Y.).

[454]  Chen Z, Zang J, Whetstine J (2006). Structural insights into histone
demethylation by JMJD2 family members. Cell.

[455]  Ng SS, Kavanagh KL, McDonough M a (2007). Crystal structures of
histone demethylase JMJD2A reveal basis for substrate specificity. Nature.

[456]  Chen Z, Zang J, Kappler J (2007). Structural basis of the recognition
of a methylated histone tail by JMJD2A. Proc Nat Acad Sci USA.

[457]  Couture J-F, Collazo E, Ortiz-Tello P a, Brunzelle JS, Trievel RC (2007). Specificity and mechanism of JMJD2A, a trimethyllysine-specific
histone demethylase. Nature structural Mol Biol.

[458]  Lee J, Thompson JR, Botuyan MV, Mer G (2008). Distinct binding modes
specify the recognition of methylated histones H3K4 and H4K20
by JMJD2A-tudor. Nature structural Mol Biol.

[459]  Rose NR, Woon ECY, Kingham GL (2010). Selective inhibitors of
JMJD2 histone demethylases: combined nondenaturing mass spectrometric
screening and crystallographic approaches. J medicinal Chem.

[460]  Chowdhury R, Yeoh KK, Tian YM (2011). The oncometabolite 2-
hydroxyglutarate inhibits histone lysine demethylases. EMBO reports.

[461]  King ONF, Li XS, Sakurai M (2010). Quantitative high-throughput
screening identifies 8-hydroxyquinolines as cell-active histone demethylase
inhibitors. PloS one.

[462]  Chang K-H, King ONF, Tumber A (2011). Inhibition of histone
demethylases by 4-carboxy-2,2’-bipyridyl compounds. ChemMedChem.

[463]  Woon ECY, Tumber A, Kawamura A (2012). Linking of 2-
oxoglutarate and substrate binding sites enables potent and highly
selective inhibition of JmjC histone demethylases. Angewandte Chemie (International ed. in English).

[464]  Koehler C, Bishop S, Dowler EF (2008). Backbone and sidechain
1H, 13C and 15N resonance assignments of the Bright/ARID domain
from the human JARID1C (SMCX) protein. Biomolecular
NMR assignments.

[465]  Tu S, Teng Y-C, Yuan C (2008). The ARID domain of the H3K4
demethylase RBP2 binds to a DNA CCGCCC motif. Nature structural
Mol Biol.

[466]  Wang GG, Song J, Wang Z (2009). Haematopoietic malignancies
caused by dysregulation of a chromatin-binding PHD finger. Nature.

[467]  Kusunoki H, Takeuchi T, Kohno T (2009). Solution structure of the ATrich
interaction domain of Jumonji/JARID2. Proteins.

[468]  Kim J, Guermah M, McGinty RK (2009). RAD6-Mediated transcription-
coupled H2B ubiquitylation directly stimulates H3K4 methylation
in human cells. Cell.

[469]  Lee J-S, Shukla A, Schneider J (2007). Histone crosstalk between
H2B monoubiquitination and H3 methylation mediated by COMPASS. Cell.

[470]  Stoleru D, Peng Y, Agosto J, Rosbash M (2004). Coupled oscillators control
morning and evening locomotor behaviour of Drosophila. Nature.

[471]  Seeler J-S, Dejean A (2003). Nuclear and unclear functions of SUMO. Nat Rev Molecular cell Biol.

[472]  Shiio Y, Eisenman RN (2003). Histone sumoylation is associated with
transcriptional repression. Proc Nat Acad Sci USA.

[473]  Chasapis CT, Spyroulias GA (2009). RING finger E(3) ubiquitin ligases:
structure and drug discovery. Curr Pharm Des.

[474]  Bacik J-P, Walker JR, Ali M, Schimmer AD, Dhe-Paganon S (2010). Crystal structure of the human ubiquitin-activating enzyme 5
(UBA5) bound to ATP: mechanistic insights into a minimalistic E1
enzyme. J Biol Chem.

[475]  Goldenberg SJ, Marblestone JG, Mattern MR, Nicholson B (2010). Strategies
for the identification of ubiquitin ligase inhibitors. Biochemical
Society transactions.

[476]  Goldenberg SJ, McDermott JL, Butt TR, Mattern MR, Nicholson 
B (2008). Strategies for the identification of novel inhibitors of deubiquitinating
enzymes. Biochem Soc transactions.

[477]  Lydeard JR, Harper JW (2010). Inhibitors for E3 ubiquitin ligases. Nature
biotechnology.

[478]  Cohen P, Tcherpakov M (2010). Will the ubiquitin system furnish as many
drug targets as protein kinases?. Cell.

[479]  Nalepa G, Rolfe M, Harper JW (2006). Drug discovery in the ubiquitinproteasome system. Nat Rev Drug Discov.

[480]  Messner S, Hottiger MO (2011). Histone ADP-ribosylation in DNA repair,
replication and transcription. Trends in cell Biol.

[481]  Hottiger MO (2011). ADP-ribosylation of histones by ARTD1: an additional
module of the histone code?. FEBS letters.

[482]  Dani N, Barbosa AJM, Del Rio A, Di Girolamo M (2013). ADPribosylated
proteins as old and new targets for anticancer therapy. Curr Pharm Des.

[483]  Laing S, Unger M, Koch-Nolte F, Haag F (2011). ADP-ribosylation of arginine. Amino acids.

[484]  Hottiger MO, Hassa PO, Lüscher B, Schüler H, Koch-Nolte F (2010). Toward a unified nomenclature for mammalian ADPribosyltransferases. Trends Biochem Sci.

[485]  Cepeda V, Fuertes MA, Castilla J (2006). Poly(ADP-ribose) polymerase-
1 (PARP-1) inhibitors in cancer chemotherapy. Recent patents on anti-cancer drug Discov.

[486]  Jagtap P, Szabó C (2005). Poly(ADP-ribose) polymerase and the therapeutic
effects of its inhibitors. Nat Rev Drug Discov.

[487]  Quénet D, El Ramy R, Schreiber V, Dantzer F (2009). The role of
poly(ADP-ribosyl)ation in epigenetic events. The international J
biochemistry &cell Biol.

[488]  Hakmé A, Wong H-K, Dantzer F, Schreiber V (2008). The expanding field
of poly(ADP-ribosyl)ation reactions. “Protein Modifications: Beyond
the Usual Suspects” Review Series.. EMBO reports.

[489]  Mendes F, Groessl M, Nazarov A a (2011). Metal-based inhibition of poly(ADP-ribose) polymerase--the guardian angel of DNA. J medicinal Chem.

[490]  Rouleau M, Patel A, Hendzel MJ, Kaufmann SH, Poirier GG (2010). PARP inhibition: PARP1 and beyond. Nat Rev Cancer.

[491]  Krishnakumar R, Kraus WL (2010). The PARP side of the nucleus: molecular
actions, physiological outcomes, and clinical targets. Molecular cell.

[492]  Kraus WL (2008). Transcriptional control by PARP-1: chromatin modulation,
enhancer-binding, coregulation, and insulation. Curr opinion in cell Biol.

[493]  Faraone-Mennella MR (2005). Chromatin architecture and functions: the
role(s) of poly(ADP-RIBOSE) polymerase and
poly(ADPribosyl)ation of nuclear proteins. Biochemistry and cell
biology = Biochimie et biologie cellulaire.

[494]  Sala A, Corona DFV (2009). Poly-ADP-ribose (PAR) as an epigenetic flag. Epigenetics: official J DNA Methylation Society.

[495]  Hassa PO, Haenni SS, Elser M, Hottiger MO (2006). Nuclear ADPribosylation
reactions in mammalian cells: where are we today and
where are we going?. Microbiology and molecular biology reviews: MMBR.

[496]  Ferraris DV (2010). Evolution of poly(ADP-ribose) polymerase-1 (PARP-
1) inhibitors. From concept to clinic. J medicinal Chem.

[497]  Guha M (2011). PARP inhibitors stumble in breast cancer. Nature biotechnology.

[498]  Dani N, Mayo E, Stilla A (2011). Mono-ADP-ribosylation of the G
protein betagamma dimer is modulated by hormones and inhibited
by Arf6. J Biol Chem.

[499]  Dani N, Stilla A, Marchegiani A (2009). Combining affinity purification
by ADP-ribose-binding macro domains with mass spectrometry
to define the mammalian ADP-ribosyl proteome. Proc Nat Acad Sci USA.

[500]  Di Girolamo M, Dani N, Stilla A, Corda D (2005). Physiological relevance
of the endogenous mono(ADP-ribosyl)ation of cellular proteins. The FEBS J.

[501]  Waaler J, Machon O, Tumova L (2012). A novel tankyrase inhibitor
decreases canonical Wnt signaling in colon carcinoma cells and reduces
tumor growth in conditional APC mutant mice. Cancer Res.

[502]  Wahlberg E, Karlberg T, Kouznetsova E (2012). Family-wide
chemical profiling and structural analysis of PARP and tankyrase
inhibitors. Nature biotechnology.

[503]  Narwal M, Venkannagari H, Lehtiö L (2012). Structural basis of selective
inhibition of human tankyrases. J medicinal Chem.

[504]  Caiafa P, Guastafierro T, Zampieri M (2009). Epigenetics: poly(ADPribosyl)
ation of PARP-1 regulates genomic methylation patterns. FASEB journal: official publication of the Federation of American
Societies for Experimental Biol.

[505]  Caiafa P (2006). Parp and epigenetic regulation. Poly (ADP-Ribosyl) ation.

[506]  Tulin A, Spradling A (2003). Chromatin loosening by poly(ADP)-ribose
polymerase (PARP) at Drosophila puff loci. Science (New York N.Y.).

[507]  Rouleau M, Aubin RA, Poirier GG (2004). Poly(ADP-ribosyl)ated chromatin
domains: access granted. J cell science.

[508]  Krishnakumar R, Gamble MJ, Frizzell KM, Berrocal JG, Kininis M, Kraus WL (2008). Reciprocal binding of PARP-1 and histone H1 at
promoters specifies transcriptional outcomes. Science (New York N.Y.).

[509]  Rogakou EP, Pilch DR, Orr AH, Ivanova VS, Bonner WM (1998). DNA
double-stranded breaks induce histone H2AX phosphorylation on
serine 139. J Biol chemistry.

[510]  Xu Y, Price BD (2011). Chromatin dynamics and the repair of DNA double strand breaks. Cell Cycle.

[511]  Fischle W, Tseng BS, Dormann HL (2005). Regulation of HP1-
chromatin binding by histone H3 methylation and phosphorylation. Nature.

[512]  Garcia B a, Joshi S, Thomas CE (2006). Comprehensive phosphoprotein
analysis of linker histone H1 from Tetrahymena thermophila. Molecular &cellular proteomics: MCP.

[513]  Lau PNI, Cheung P (2011). Histone code pathway involving H3 S28
phosphorylation and K27 acetylation activates transcription and antagonizes
polycomb silencing. Proc Nat Acad Sci USA.

[514]  Simboeck E, Sawicka A, Zupkovitz G (2010). A phosphorylation
switch regulates the transcriptional activation of cell cycle regulator
p21 by histone deacetylase inhibitors. J Biol Chem.

[515]  Banerjee T, Chakravarti D (2011). A peek into the complex realm of histone
phosphorylation. Molecular and cellular Biol.

[516]  Xhemalce B, Dawson MA, Bannister AJ (2006). Histone Modifications. In: Encyclopedia of Molecular Cell Biology and Molecular Medicine.

[517]  Tan E, Besant PG, Zu XL (2004). Histone H4 histidine kinase displays
the expression pattern of a liver oncodevelopmental marker. Carcinogenesis.

[518]  Besant PG, Attwood PV (2010). Histidine phosphorylation in histones and
in other mammalian proteins. Methods in enzymology.

[519]  Kaleem A, Hoessli DC, Ahmad I (2008). Immediate-early gene
regulation by interplay between different post-translational modifications
on human histone H3. J Cellular Biochem.

[520]  Ahmad W, Shabbiri K, Nazar N, Nazar S, Qaiser S, Shabbir Mughal MA (2011). Human linker histones: interplay between phosphorylation
and O-??-GlcNAc to mediate chromatin structural modifications. Cell division.

[521]  Gloster TM, Vocadlo DJ (2010). Mechanism, Structure, and Inhibition of
O-GlcNAc Processing Enzymes. Curr signal transduction therapy.

[522]  Hanover J a (2010). Epigenetics Gets Sweeter: O-GlcNAc Joins the “Histone Code. Chemistry &Biol.

[523]  Hanover J a (2012). Krause MW, Love DC. Post-translational modifications:
Bittersweet memories: linking metabolism to epigenetics
through O-GlcNAcylation. Nat Rev Molecular Cell Biol.

[524]  Sakabe K, Wang Z, Hart GW (2010). Beta-N-acetylglucosamine (OGlcNAc)
is part of the histone code. Proc Nat Acad Sci USA.

[525]  Zhang S, Roche K, Nasheuer H-P, Lowndes NF (2011). Modification of
histones by sugar ??-N-acetylglucosamine (GlcNAc) occurs on mul612
tiple residues, including histone H3 serine 10, and is cell cycleregulated. J Biol Chem.

[526]  Fujiki R, Hashiba W, Sekine H (2011). GlcNAcylation of histone
H2B facilitates its monoubiquitination. Nature.

[527]  Sinclair DAR, Syrzycka M, Macauley MS (2009). Drosophila OGlcNAc
transferase (OGT) is encoded by the Polycomb group
(PcG) gene, super sex combs (sxc). Proc Nat Acad Sci USA.

[528]  Dall’Olio F, Malagolini N, Trinchera M, Chiricolo M (2012). Mechanisms
of cancer-associated glycosylation changes. Frontiers in bioscience:
a journal and virtual library.

[529]  Caretti A, Sirchia SM, Tabano S, Zulueta A, Dall’Olio F, Trinchera 
M (2012). DNA methylation and histone modifications modulate the ??1,3
galactosyltransferase ??3Gal-T5 native promoter in cancer cells. The international J biochemistry &cell Biol.

[530]  Dall’olio F, Malagolini N, Chiricolo M (2011). Glycosylation in Cancer. Carbohydrate Chem.

[531]  Slawson C, Hart GW (2011). O-GlcNAc signalling: implications for cancer cell biology. Nat Rev Cancer.

[532]  Li M, Song L, Qin X (2010). Glycan changes: cancer metastasis and anticancer vaccines. J Biosciences.

[533]  Dall’olio F, Vanhooren V, Chen CC, Slagboom PE, Wuhrer M, Franceschi C (2012). N-glycomic biomarkers of biological aging and longevity: A link with inflammaging. Ageing research reviews.

[534]  Jínek M, Rehwinkel J, Lazarus BD, Izaurralde E, Hanover JA, Conti E (2004). The superhelical TPR-repeat domain of O-linked GlcNAc
transferase exhibits structural similarities to importin alpha. Nature structural Mol Biol.

[535]  Jiang J, Lazarus MB, Pasquina L, Sliz P, Walker S (2012). A neutral
diphosphate mimic crosslinks the active site of human O-GlcNAc
transferase. Nature chemical Biol.

[536]  Lazarus MB, Nam Y, Jiang J, Sliz P, Walker S (2011). Structure of human
O-GlcNAc transferase and its complex with a peptide substrate. Nature.

[537]  Jakeman DL (2011). Mechanisms of glycosyltransferases: the in and the out. Chembiochem: a Eur J chemical Biol.

[538]  Roychoudhury R, Pohl NLB (2010). New structures, chemical functions,
and inhibitors for glycosyltransferases. Cur opinion Chem Biol.

[539]  Hosoguchi K, Maeda T, Furukawa JI (2010). An efficient approach
to the discovery of potent inhibitors against glycosyltransferases. J medicinal Chem.

[540]  Agard NJ, Bertozzi CR (2009). Chemical approaches to perturb profile
and perceive glycans. Accounts of chemical Res.

[541]  Eskandari R, Kuntz D a, Rose DR, Pinto BM (2010). Potent glucosidase
inhibitors: de-O-sulfonated ponkoranol and its stereoisomer. Organic
letters.

[542]  Asano N (2009). Sugar-mimicking glycosidase inhibitors: bioactivity and
application. Cellular and molecular life sciences: CMLS.

[543]  Hinou H, Nishimura S-ichiro (2009). Mechanism-based probing, characterization,
and inhibitor design of glycosidases and glycosyltransferases. Curr Topics Med Chem.

[544]  Patel SM, de la Fuente M, Ke S (2011). High throughput discovery
of heteroaromatic-modifying enzymes allows enhancement of novobiocin
selectivity. Chemical communications (Cambridge, England).

[545]  Stubbs K a, Zhang N, Vocadlo DJ (2006). A divergent synthesis of 2-acyl
derivatives of PUGNAc yields selective inhibitors of OGlcNAcase. Organic &biomolecular Chem.

[546]  Dorfmueller HC, Borodkin VS, Schimpl M, Shepherd SM, ShpiroN a, van Aalten DMF (2006). GlcNAcstatin: a picomolar, selective OGlcNAcase
inhibitor that modulates intracellular O-glcNAcylation
levels. J American Chemical Society.

[547]  Macauley MS, Vocadlo DJ (2010). Increasing O-GlcNAc levels: An overview
of small-molecule inhibitors of O-GlcNAcase. Biochimica et
biophysica acta.

[548]  Gross BJ, Kraybill BC, Walker S (2005). Discovery of O-GlcNAc transferase
inhibitors. J American Chemical Society.

[549]  Doyle K, Fitzpatrick F a (2010). Redox signaling, alkylation (carbonylation)
of conserved cysteines inactivates class I histone deacetylases
1, 2, and 3 and antagonizes their transcriptional repressor function. J Biol Chem.

[550]  Wondrak GT, Cervantes-Laurean D, Jacobson EL, Jacobson MK (2000). Histone carbonylation in vivo and in vitro. The Biochemical J.

[551]  García-Giménez JL, Ledesma AMV, Esmoris I (2012). Histone carbonylation occurs in proliferating cells. Free radical biology &medicine.

[552]  Nakamura A, Kawakami K, Kametani F, Nakamoto H, Goto S (2010). Biological significance of protein modifications in aging and calorie
restriction. Annals of the New York Academy of Sciences.

[553]  Anzilotti C, Pratesi F, Tommasi C, Migliorini P (2010). Peptidylarginine
deiminase 4 and citrullination in health and disease. Autoimmunity reviews.

[554]  Cuthbert GL, Daujat S, Snowden AW (2004). Histone deimination antagonizes arginine methylation. Cell.

[555]  Wang Y, Wysocka J, Sayegh J (2004). Human PAD4 regulates histone
arginine methylation levels via demethylimination. Science (New York, N.Y.).

[556]  Chang X, Fang K (2010). PADI4 and tumourigenesis. Cancer cell international.

[557]  Tanikawa C, Espinosa M, Suzuki A (2012). Regulation of histone
modification and chromatin structure by the p53-PADI4 pathway. Nature communications.

[558]  Wang Y, Li P, Wang S (2012). Anticancer PAD inhibitors regulate
the autophagy flux and the mammalian target of rapamycin complex
1 activity. J Biol Chem.

[559]  Luo Y, Arita K (2006). Inhibitors and Inactivators of Protein Arginine
Deiminase 4: Functional and Structural Characterization. Biochemistry.

[560]  Causey CP, Jones JE, Slack JL (2011). The development of N-??-(2-
carboxyl)benzoyl-N(5)-(2-fluoro-1-iminoethyl)-l-ornithine amide
(o-F-amidine) and N-??-(2-carboxyl)benzoyl-N(5)-(2-chloro-1-
iminoethyl)-l-ornithine amide (o-Cl-amidine) as second generation
protein arginine deiminase (PAD) inhibit. J medicinal Chem.

[561]  Jones JE, Slack JL, Fang P (2012). Synthesis and screening of a
haloacetamidine containing library to identify PAD4 selective inhibitors. ACS chemical Biol.

[562]  Horikoshi N, Tachiwana H, Saito K (2011). Structural and biochemical
analyses of the human PAD4 variant encoded by a functional
haplotype gene. Acta crystallographica. Section D, Biological crystallography.

[563]  Arita K, Shimizu T, Hashimoto H, Hidaka Y, Yamada M, Sato M (2006). Structural basis for histone N-terminal recognition by human peptidylarginine
deiminase 4. Proc Nat Acad Sci USA.

[564]  Hymes J, Fleischhauer K, Wolf B (1995). Biotinylation of histones by
human serum biotinidase: assessment of biotinyl-transferase activity
in sera from normal individuals and children with biotinidase
deficiency. Biochemical and molecular medicine.

[565]  Pestinger V, Wijeratne SSK, Rodriguez-Melendez R, Zempleni J (2011). Novel histone biotinylation marks are enriched in repeat regions
and participate in repression of transcriptionally competent genes. J
nutritional bioChem.

[566]  Kothapalli N, Camporeale G, Kueh A (2005). Biological functions of
biotinylated histones. J nutritional bioChem.

[567]  Hassan YI, Zempleni J (2008). A novel, enigmatic histone modification:
biotinylation of histones by holocarboxylase synthetase. Nutrition
reviews.

[568]  Filenko N a, Kolar C, West JT (2011). The role of histone H4 biotinylation
in the structure of nucleosomes. PloS one.

[569]  Chew YC, West JT, Kratzer SJ (2008). Biotinylation of histones
represses transposable elements in human and mouse cells and cell
lines and in Drosophila melanogaster. J nutrition.

[570]  Kobza K, Camporeale G, Rueckert B (2005). K4, K9 and K18 in
human histone H3 are targets for biotinylation by biotinidase. The FEBS J.

[571]  Camporeale G, Shubert EE, Sarath G, Cerny R, Zempleni J (2004). K8 and
K12 are biotinylated in human histone H4. Eur J biochemistry 
FEBS.

[572]  Bagautdinov B, Kuroishi C, Sugahara M, Kunishima N (2005). Crystal
structures of biotin protein ligase from Pyrococcus horikoshii OT3
and its complexes: structural basis of biotin activation. J Mol Biolo.

[573]  Bagautdinov B, Matsuura Y, Bagautdinova S, Kunishima N (2008). Protein
biotinylation visualized by a complex structure of biotin protein
ligase with a substrate. J Biol Chem.

[574]  Santos-Rosa H, Kirmizis A, Nelson C (2009). Histone H3 tail clipping
regulates gene expression. Nature structural Mol Biol.

[575]  Nelson CJ, Santos-Rosa H, Kouzarides T (2006). Proline isomerization of
histone H3 regulates lysine methylation and gene expression. Cell.

[576]  Fabbri M, Calin G a (2010). Epigenetics and miRNAs in human cancer.

[577]  O’Connell RM (2012). MicroRNAs function on a new level. Blood.

[578]  Benhamed M, Herbig U, Ye T, Dejean A, Bischof O (2012). Senescence is
an endogenous trigger for microRNA-directed transcriptional gene
silencing in human cells. Nature cell Biol.

[579]  Lopez-Serra P, Esteller M (2011). DNA methylation-associated silencing
of tumor-suppressor microRNAs in cancer. Oncogene.

[580]  Cho WCS (2011). Grand Challenges and Opportunities in Deciphering the
Role of Non-Coding RNAs in Human Diseases. Frontiers in genetics.

[581]  Cho WC (2012). Exploiting the therapeutic potential of microRNAs in
human cancer. Exp opinion on therapeutic targets.

[582]  Garzon R, Marcucci G, Croce CM (2010). Targeting microRNAs in cancer:
rationale, strategies and challenges. Nat Rev Drug discov.

[583]  Iorio MV, Croce CM (2009). MicroRNAs in cancer: small molecules with a huge impact. J clinical oncology: official J Am Soc Clin Oncoly.

[584]  Parker JS (2010). How to slice: snapshots of Argonaute in action. Silence.

[585]  Bouasker S, Simard MJ (2009). Structural biology: Tracing Argonaute
binding. Nature.

[586]  Hutvagner G, Simard MJ (2008). Argonaute proteins: key players in RNA
silencing. Nat Rev Mol Cell Biol.

[587]  Tan GS, Chiu C-H, Garchow BG, Metzler D, Diamond SL, Kiriakidou M (2012). Small molecule inhibition of RISC loading. ACS Chem Biol.

[588]  Kasinski AL, Slack FJ (2011). Epigenetics and genetics. MicroRNAs en
route to the clinic: progress in validating and targeting microRNAs
for cancer therapy. Nat Rev Cancer.

[589]  Chen X-ping, Du G-hua (2007). Target validation??: A door to drug discovery. Drug Discov Ther.

[590]  Flemming A (2009). Chemoinformatics: Where “magic bullets” go astray. Nat Rev Drug discov.

[591]  Bottegoni G, Favia AD, Recanatini M, Cavalli A (2012). The role of
fragment-based and computational methods in polypharmacology. Drug Discov Today.

[592]  Petrelli A, Giordano S (2008). From single- to multi-target drugs in cancer
therapy: when aspecificity becomes an advantage. Curr Med Chem.

[593]  vel Szic KS, Ndlovu MN, Haegeman G, Vanden Berghe W (2010). Nature
or nurture: let food be your epigenetic medicine in chronic inflammatory
disorders. Biochem Pharmacol.

[594]  Beher D, Wu J, Cumine S (2009). Resveratrol is not a direct activator
of SIRT1 enzyme activity. Chem Biol Drug Des.

[595]  Denu JM (2012). Fortifying the Link between SIRT1, Resveratrol, and
Mitochondrial Function. Cell Metabol.

[596]  Price NL, Gomes AP, Ling AJY (2012). SIRT1 Is Required for
AMPK Activation and the Beneficial Effects of Resveratrol on Mitochondrial
Function. Cell Metabolism.

[597]  Moniot S, Weyand M, Steegborn C (2012). Structures, substrates, and
regulators of Mammalian sirtuins - opportunities and challenges for
drug development. Frontiers in pharmacology.

[598]  Han Z, Xing X, Hu M, Zhang Y, Liu P, Chai J (2007). Structural basis of
EZH2 recognition by EED. Structure (London, England􀀁: 1993).

[599]  Barbosa AJM, Del Rio A (2012). Freely accessible databases of commercial
compounds for high- throughput virtual screenings. Curr Topics Med Chem.

[600]  Del Rio A, Barbosa AJM, Caporuscio F, Mangiatordi GF (2010). Co-
CoCo: a free suite of multiconformational chemical databases for
high-throughput virtual screening purposes. Molecular bioSystems.

[601]  Del Rio a, Barbosa A, Caporuscio F (2011). Use of large multiconformational
databases with structure-based pharmacophore models for
fast screening of commercial compound collections. J Cheminformatics.

[602]  Sanders MPA, Barbosa AJM, Zarzycka B (2012). A comparative
analysis of pharmacophore screening tools. J Chem Information Modeling.

[603]  Heinke R, Carlino L, Kannan S, Jung M, Sippl W (2011). Computer- and structure-based lead design for epigenetic targets. Bioorganic Med Chem.

[604]  Keiser MJ, Setola V, Irwin JJ (2009). Predicting new molecular
targets for known drugs. Nature.

[605]  Chong CR, Sullivan DJ (2007). New uses for old drugs. Nature.

[606]  Harvey AL (2007). Natural products as a screening resource. Cur opinion Chem Biol.

[607]  Harvey AL (2008). Natural products in drug discovery. Drug Discov Today.

[608]  Swinne  DC, Anthony J (2011). How were new medicines discovered?. Nat Rev Drug Discov.

[609]  Kotz J (2012). Phenotypic screening, take two. Science-Business eXchange.

